# Bacterial meningitis: Aetiology, risk factors, disease trends and severe sequelae during 50 years in Sweden

**DOI:** 10.1111/joim.13488

**Published:** 2022-04-03

**Authors:** Nils Block, Pontus Naucler, Philippe Wagner, Eva Morfeldt, Birgitta Henriques‐Normark

**Affiliations:** ^1^ Department of Microbiology, Tumor and Cell biology (MTC) Biomedicum, Karolinska Institutet Stockholm Sweden; ^2^ Department of Infectious Diseases Visby County Hospital Visby Sweden; ^3^ Division of Infectious Diseases Department of Medicine Solna Karolinska Institutet Stockholm Sweden; ^4^ Department of Infectious Diseases Karolinska University Hospital Stockholm Sweden; ^5^ Centre for Clinical Research Västmanland Västmanland County Hospital Uppsala University Västerås Sweden; ^6^ Public Health Agency of Sweden Solna Sweden; ^7^ Department of Clinical Microbiology Karolinska University Hospital Stockholm Sweden

**Keywords:** bacterial meningitis, conjugate vaccines, *Haemophilus influenzae*, severe sequelae, *Streptococcus pneumoniae*

## Abstract

**Background:**

Bacterial meningitis (BM) is a rare but severe infection. Few population‐based studies have characterised BM episodes and sequelae over long periods.

**Methods:**

This was a population‐based observational cohort study with national coverage, using data on aetiological pathogens, sex, premorbid conditions, steroid pretreatment, severe sequelae and birth, death and diagnosis dates collected from 10,339 patients with BM reported to the National Board of Health and Welfare in Sweden between 1964 and 2014.

**Results:**

During the 50‐year study period, the incidence of BM decreased in young children, but not in the elderly. The most common cause of BM was pneumococci (34%), followed by *Haemophilus influenzae* (26%), and meningococci (18%), mainly community acquired. Premorbid conditions were found in 20%. After the *H. influenzae* type b vaccine was introduced in 1993, the BM incidence decreased by 36%. Following pneumococcal conjugated vaccine introduction in 2009, the incidence and 30‐day mortality from pneumococcal meningitis decreased by 64% and 100%, respectively, in previously healthy children, and the 30‐day mortality decreased by 64% among comorbid adults. The BM incidence in immunosuppressed patients increased by 3% annually post vaccine introduction. The 30‐day mortality was 3% in children and 14% in adults, and the rate of severe sequelae was 44%. On average, patients lost 11 years of healthy life due to BM.

**Conclusion:**

The introduction of conjugated vaccines into the childhood vaccination program has reduced the incidence of BM in young children, but not in adults. Post vaccine introduction, patients present with more premorbid conditions and other bacterial causes of BM, emphasising the need for a correct diagnosis when treating these infections.

## Graphical abstract

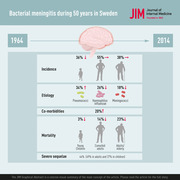

**Abbreviations**
BM: bacterial meningitis
CBM: community‐acquired BM
CI95%: 95% confidence interval
CNS: central nervous system
HBM: healthcare‐associated BM
HBMF: healthcare‐associated BM facility
Hib: *Haemophilus influenzae* type b vaccine
ICD: International Classification of Diseases
OR: odds ratio
PCV: pneumococcal conjugate vaccine
RBM: recurrent bacterial meningitis

## Introduction

Bacterial meningitis (BM) is a rare infection that can be complicated by severe sequelae [1]. Infection occurs among patients of all ages, who may have premorbid health conditions, in both community and healthcare settings [2]. Most patients have a single episode; rarely, the infection is recurrent [3]. Community‐acquired BM (CBM) is distinct from healthcare‐associated BM (HBM). HBM has recently been associated with healthcare facilities (HBMF), such as hospitals or nursing homes, and with clinical procedures resulting in post‐neurosurgical infection, with or without an implanted, indwelling intracranial device [4, 5, 6]. Vaccines targeting BM‐causing pathogens have been introduced in several countries. In Sweden, the *Haemophilus influenzae* type b vaccine (Hib) was introduced into the childhood vaccination program in 1993, and pneumococcal conjugate vaccines (PCVs) were launched nationally in 2009. The effects of vaccine introduction on BM and its severe sequelae remain to be determined, and patient groups with an increased risk of BM need to be identified for preventive measures. During the conjugate vaccine era, overall age‐specific survival in Sweden has increased, in part due to new medical treatments for chronic diseases [7, 8, 9].

Here, we studied the incidence and aetiology of BM, and its associated premorbid conditions, 30‐day mortality and risk factors for severe sequelae among patients with BM over 50 years in Sweden. Additionally, the importance of specific predisposing conditions for recurrent BM (RBM) was investigated.

## Methods

### Data collection

We performed a 50‐year population‐based cohort study with national coverage of BM in Sweden. Patients diagnosed with pathogen‐specified BM prospectively reported to the National Patient Register (NPR) using the Swedish version of the International Classification of Diseases (ICD) 7–10 were identified from the registers of the National Board of Health and Welfare. Data, including their aetiologic pathogens, date of birth, admission, discharge, death, migration, severe sequelae, clinical setting and premorbid conditions, were collected. The NPR contains Swedish inpatient data from 1964 onwards, with national coverage from 1987. National Swedish registers for open specialist care (2001 onwards) and pharmacology (July 2005 onwards) provided complementary information regarding severe sequelae and pharmacological pre‐admission treatment, respectively, for patients diagnosed after these dates. This study was approved by the Uppsala local ethics committee.

### Definitions

BM was defined as an episode of pathogen‐specified BM identifiable using ICD 9/10 codes, as used in Swedish health care from 1987 onwards. The ICD codes were provided by the attending physicians and bacteria were mainly detected in the cerebrospinal fluid. Detailed definitions of age groups, Charlson comorbidities, risk factors for BM, pharmacological immunosuppression, HBM and pathogens, including ICD codes and classification as specific predisposing conditions, are available in the Supplementary Information (Materials and Methods, Tables S1 and S2). To minimise potential bias due to different ICD versions, coding practices and subnational coverage, no statistical analyses were performed on patient data from before 1987. To further test data validity, we collected 46 medical records of consecutive BM episodes from different Swedish hospitals (Table S3). RBM was defined as a subsequent separate BM episode with the same or different pathogens as in the index episode. Detailed definitions of the RBM are available in the Supplementary Information, Materials and Methods. Severe sequelae were defined as death within 30 days or new severe neurological sequelae in hospitals within either 90 days of admission, for acute cerebrovascular events, or 1 year of admission, for other severe neurological sequelae (hydrocephalus, epilepsy, paresis of one or more limbs, loss of vision and/or other cranial nerve dysfunctions excluding hearing impairment, sensorineural hearing impairment, depression with antidepressant pharmacological treatment, moderate and severe anxiety or attention deficit hyperactivity disorder) [1, 10, 11]. Pharmacological data were available from 2005 onwards only; therefore, data on severe sequelae were restricted to episodes reported thereafter. Detailed definitions of severe sequelae and disease burden are provided in the Supplementary Information.

### Statistical analysis

Logistic regression was used to analyse categorical data. Negative binomial regression with robust estimation of standard errors was used to analyse count data and for survival analysis with clustering of patients, as previously described [12, 13, 14]. The incidence rates for BM were calculated using age‐specific data on the midyear population from Statistics Sweden. Average percentage changes were derived through exponentiation of the regression coefficients and their 95% confidence intervals from the regression analysis. Statistical significance was set at *p* < 0.05. All statistical analyses were performed using STATA16.

## Results

### Patient characteristics and aetiology of BM

A total of 10,771 episodes of BM met the inclusion criteria from 1965 to 2014. Thirty‐six percent (3859/10,771) of all episodes occurred in children under 5 years, of which 8% (303/3859) were neonatal cases. Fifty‐five percent (5894/10,771) were adults (≥18 years), among whom the elderly (≥65 years) constituted 38% (2233/5894), corresponding to 21% (2233/10,771) of all patients (Table 1). Fifty‐three percent were females (5673/10,771). The most common bacterial cause of BM during the period 1965–2014 was *Streptococcus pneumoniae* (pneumococci, 34%), followed by *Haemophilus influenzae* (*Haemophilus*, 26%), *Neisseria meningitidis* (meningococci, 18%), *Staphylococcus* spp. (staphylococci, 9%), non‐pneumococcal streptococci (streptococci, 8%), *Listeria monocytogenes* (*Listeria*, 3%) and gram‐negative bacteria, excluding *Haemophilus* and meningococci (1%) (Table 1). In 0.4% of the cases (40/10,771), multiple species were found. Aetiology differed between the age groups. In the youngest children (0–4 years), *Haemophilus* was the most prominent cause, found in 56% (2172/3859), followed by pneumococci (17%, *n* = 669), meningococci (13%, *n* = 508) and streptococci (9%, *n* = 365). In contrast, among the elderly, pneumococci dominated, with 48% (2835/5894), followed by meningococci (15%), staphylococci (14%), streptococci (8%), *Haemophilus* (7%) and *Listeria* (5%).

**Table 1:** Cohort characteristics, bacterial meningitis, Sweden 1965–2014

Characteristic	*Haemophilus influenzae, n *= 2773, *n* (%)	*Streptococcus pneumoniae, n* = 3708, *n* (%)	*Neisseria meningitidis, n* = 1935, *n* (%)	Non‐pneumococcal streptococci, *n* = 903, *n* (%)	Gram‐negatives, *n* = 143, *n* (%)	*Listeria monocytogenes, n* = 349, *n* (%)	*Staphylococcus* spp., *n* = 920, *n* (%)	Polymicrobial, *n* = 40, *n* (%)	Bacterial meningitis, *n* = 10,771, *n* (%)
**Age 0–4 years**	2172 (78)	669 (18)	508 (26)	365 (40)	51 (36)	15 (4)	74 (8)	5 (12)	3859 (36)
Neonatal	17 (1)	12 (0)	3 (0)	221 (24)	34 (24)	4 (1)	12 (1)	0 (0)	303 (3)
Non‐neonatal	2155 (78)	657 (18)	505 (26)	144 (16)	17 (12)	11 (3)	62 (7)	5 (12)	3556 (33)
**Age 5–17 years**	162 (6)	204 (6)	553 (29)	45 (5)	5 (3)	11 (3)	35 (4)	3 (8)	1018 (9)
**Age 18–64 years**	285 (10)	1639 (44)	768 (40)	272 (30)	47 (33)	115 (33)	510 (55)	25 (62)	3661 (34)
**Age 65 years or older**	154 (6)	1196 (32)	106 (5)	221 (24)	40 (28)	208 (60)	301 (33)	7 (18)	2233 (21)
**Male gender**	1367 (49)	1737 (47)	907 (47)	444 (49)	55 (38)	152 (44)	421 (46)	15 (38)	5098 (47)
**Community‐acquired BM**	2679 (97)	3341 (90)	1837 (95)	568 (63)	66 (46)	241 (69)	448 (49)	31 (78)	9211 (86)
**Healthcare‐associated BM**	94 (3)	367 (10)	98 (5)	335 (37)	77 (54)	108 (31)	472 (51)	9 (22)	1560 (14)
Healthcare‐facility acquired^a^	74 (3)	287 (8)	85 (4)	282 (31)	52 (36)	100 (29)	183 (20)	6 (15)	1069 (10)
Nursing‐home acquired	5 (0)	17 (0)	3 (0)	7 (1)	1 (1)	5 (1)	6 (1)	0 (0)	44 (0)
Hospital acquired	69 (2)	270 (7)	82 (4)	275 (30)	51 (36)	95 (27)	177 (19)	6 (15)	1025 (10)
Post‐neurosurgical	20 (1)	80 (2)	13 (1)	53 (6)	25 (17)	8 (2)	289 (31)	3 (8)	491 (5)
No indwelling device present	12 (0)	55 (1)	12 (1)	40 (4)	12 (8)	5 (1)	203 (22)	3 (8)	342 (3)
Indwelling device present	8 (0)	25 (1)	1 (0)	13 (1)	13 (9)	3 (1)	86 (9)	0 (0)	149 (1)
Intracranial shunt	7 (0)	19 (1)	1 (0)	13 (1)	13 (9)	3 (1)	85 (9)	0 (0)	141 (1)
Intracranial electrode	0 (0)	0 (0)	0 (0)	0 (0)	0 (0)	0 (0)	1 (0)	0 (0)	1 (0)
Cochlear implant	1 (0)	7 (0)	0 (0)	0 (0)	0 (0)	0 (0)	0 (0)	0 (0)	8 (0)
**Premorbid conditions**	145 (5)	889 (24)	132 (7)	220 (24)	65 (45)	203 (58)	488 (53)	15 (38)	2157 (20)
Primary immune deficiency	5 (0)	22 (1)	6 (0)	4 (0)	0 (0)	0 (0)	2 (0)	1 (2)	40 (0)
Rheumatic disease	12 (0)	96 (3)	17 (1)	16 (2)	7 (5)	48 (14)	44 (5)	1 (2)	241 (2)
Malignant neoplasia^b^	35 (1)	277 (7)	20 (1)	65 (7)	22 (15)	85 (24)	131 (14)	6 (15)	641 (6)
*Haematological*	17 (1)	120 (3)	4 (0)	18 (2)	4 (3)	53 (15)	22 (2)	4 (10)	242 (2)
*Lymphoma*	4 (0)	36 (1)	2 (0)	0 (0)	1 (1)	13 (4)	11 (1)	2 (5)	69 (1)
*Leukaemia*	4 (0)	42 (1)	2 (0)	11 (1)	3 (2)	28 (8)	9 (1)	2 (5)	101 (1)
*Plasma‐cell neoplasia*	10 (0)	48 (1)	0 (0)	8 (1)	0 (0)	18 (5)	4 (0)	0 (0)	88 (1)
*Other malignant neoplasia*	25 (1)	231 (6)	20 (1)	58 (6)	22 (15)	73 (21)	127 (14)	6 (15)	562 (5)
Lesions of the CNS^c^	50 (2)	216 (6)	17 (1)	53 (6)	31 (22)	25 (7)	257 (28)	3 (8)	652 (6)
*Parenchymal tumour* ^d^	7 (0)	14 (0)	1 (0)	12 (1)	12 (8)	1 (0)	80 (9)	0 (0)	127 (1)
*Meningeal tumour* ^d^	4 (0)	14 (0)	2 (0)	4 (0)	3 (2)	1 (0)	22 (2)	0 (0)	50 (0)
*Ischaemic CVL*	9 (0)	59 (2)	4 (0)	11 (1)	10 (7)	20 (6)	51 (6)	0 (0)	164 (2)
*Haemorrhagic CVL*	11 (0)	23 (1)	3 (0)	20 (2)	6 (4)	1 (0)	115 (12)	1 (2)	180 (2)
*Traumatic parameningeal bleed*	6 (0)	33 (1)	4 (0)	8 (1)	4 (3)	5 (1)	34 (4)	2 (5)	96 (1)
*Basilar skull fracture*	11 (0)	65 (2)	5 (0)	6 (1)	1 (1)	3 (1)	16 (2)	1 (2)	108 (1)
*Complicated skull fracture*	6 (0)	37 (1)	2 (0)	2 (0)	0 (0)	0 (0)	4 (0)	0 (0)	51 (0)
Other Charlson comorbidity	75 (3)	469 (13)	92 (5)	125 (14)	39 (27)	115 (33)	235 (26)	8 (20)	1158 (11)
**Steroid pretreatment** ^e^	8 (0)	38 (1)	3 (0)	18 (2)	7 (5)	40 (11)	31 (3)	1 (2)	146 (1)

Abbreviations: BM, bacterial meningitis; CNS, central nervous system; CVL, cerebrovascular lesion.

^a^ Also known as nosokomial infections.

^b^ Including malignant parenchymal and meningeal tumour of the central nervous system.

^c^ Premorbid traumatic, neoplastic and cerebrovascular lesions of the central nervous system (post‐neurosurgical episodes are reported under health‐care associated episodes).

^d^ Including malignant and benign tumour forms.

^e^ Pharmacological data available from July 2005, thus percentages are based on the number of episodes per pathogen from the year 2005 to the year 2014.

### Temporal trends of BM and the effects of conjugate vaccines

**Fig. 1:**
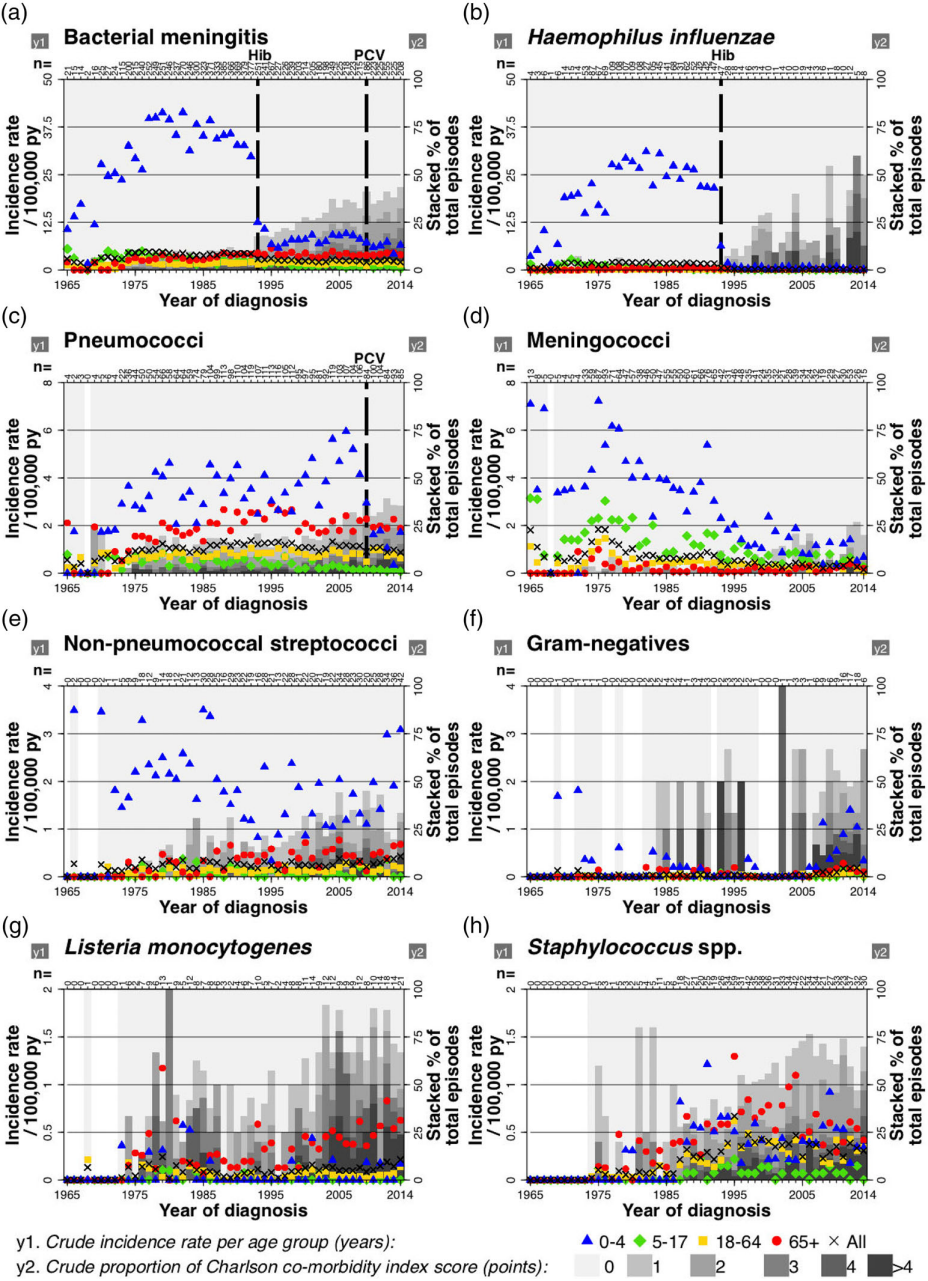
Incidence rate (IR) of and comorbidity with bacterial meningitis (BM). The annual IRs of BM in Sweden between 1965 and 2014 by single pathogen and age group are shown in relation to the introduction of H. influenzae B (Hib) and pneumococcal conjugate vaccines (PCVs) on the left y‐axis (y1) and annual stacked proportions of patient Charlson comorbidity index scores on the right y‐axis (y2) in grayscale. The total annual number of episodes is shown for each year. The annual incidence of (a) all BM, followed by that of BM due to single pathogens, namely (b) Haemophilus influenzae, (c) Streptococcus pneumoniae, (d) Neisseria meningitidis, (e) non‐pneumococcal streptococci, (f) gram‐negative bacteria, (g) Listeria monocytogenes and (h) Staphylococcal spp.

The BM incidence increased in children under 5 years from 1970 to 1980 and remained constant until the beginning of the 1990s, when it decreased dramatically (Fig. 1). *Haemophilus* was the dominant agent; however, after 1993, when the Hib vaccine was introduced, there were few Hib cases. The incidence of meningococci was also high between 1965 and the mid‐1970s in young children, but decreased thereafter, despite the absence of a meningococcal vaccine in the vaccination program (Fig. 1 and Figs S1 and S2). The incidence of episodes caused by gram‐negative bacteria and streptococci was also most prominent in young children, and there were increases in neonatal BM (annual change 6%, 95% confidence interval [CI95%] 1%–11%, *p* = 0.01) and neonatal gram‐negative BM (annual change 23%, CI95% 9%–39%, *p* = 0.001) during the conjugate‐vaccine era (Table 1, Fig. S3). Overall, *E. coli* was the most common gram‐negative bacterium among neonates (28/34), in contrast to *Pseudomonas*, which was mainly found in adults (12/14) (Table S4), *Listeria* infections were predominantly found in the elderly (208/349, 60%) and were practically absent among neonates (Table 1). The incidence of BM episodes caused by staphylococci, *Listeria* and gram‐negative bacteria increased from 0.4 to 0.6 per 100,000 person‐years (incidence rate ratio 1.8, CI95% 1.5–2.1, *p* < 0.001), corresponding to an increase among BM cases from 8% (150/1832) to 26% (299/1136) (odds ratio [OR] 4.0, CI95% 3.2–5.0, *p* < 0.001) when comparing the period 1987–1991 with 2010–2014. The incidence of pneumococcal meningitis was highest in children under 5 years, at 5.1 per 100,000 person‐years prevaccination (2004–2008), which decreased after introduction of the first PCV, PCV7, to 1.5 in the post‐vaccination period (2009–2014) (percentage change after vaccination −70%, CI95% −82 to −49, *p* < 0.001) (Fig. 1, Table 2).

**Table 2:** Incidence rate and all‐cause 30‐day mortality of pneumococcal meningitis before and after introduction of pneumococcal conjugate vaccine in 2009^a^

Characteristic	Years 2004–2008	Years 2009–2013	Percent change after vaccination (95% confidence interval)	*p*‐Value
**Incidence rate** ^b^ (*N*)				
*Children*	1.5 ± 0.2 (145)	0.5 ± 0.3 (52)	−64 (−77 to −44)	<0.001
Age 0–4 years	5.1 ± 0.7 (129)	1.5 ± 1.0 (43)	−70 (−82 to −49)	<0.001
Age 5–17 years	0.2 ± 0.1 (16)	0.1 ± 0.0 (9)	−41 (−60 to −14)	0.007
Charlson index score 0	1.4 ± 0.2 (133)	0.5 ± 0.2 (46)	−65 (−76 to −47)	<0.001
Charlson index score >0	0.1 ± 0.0 (8)	0.1 ± 0.0 (4)	1 (−31 to 47)	0.97
*Adult*	1.1 ± 0.1 (394)	1.1 ± 0.1 (413)	0 (−12 to 12)	0.97
Age 18–64 years	0.8 ± 0.1 (230)	0.8 ± 0.2 (231)	−3 (−23 to 23)	0.81
Age 65+ years	2.1 ± 0.3 (164)	2.1 ± 0.2 (182)	0 (−13 to 14)	0.96
Charlson index score 0	0.7 ± 0.1 (228)	0.7 ± 0.1 (199)	−2 (−19 to 18)	0.82
Charlson index score >0	0.4 ± 0.0 (139)	0.4 ± 0.0 (134)	3 (−6 to 13)	0.53
Community acquired	1.0 ± 0.1 (449)	0.8 ± 0.1 (372)	−20 (−30 to −10)	0.001
Healthcare‐facility acquired	0.2 ± 0.0 (94)	0.1 ± 0.0 (63)	−20 (−34 to −4)	0.02
Post‐neurosurgical	0.0 ± 0.0 (14)	0.1 ± 0.0 (30)	106 (2–319)	0.05
Total	0.9 ± 0.5 (539)	0.8 ± 0.4 (465)	−17 (−25 to −8)	<0.001
**All‐cause 30‐day mortality**,^c^ % (n/N)				
*Children*	4.3 (6/141)	0.0 (0/50)	−100 (−100 to −100)	–^d^
Age 0–4 years	4.8 (6/126)	0.0 (0/43)	−100 (−100 to −100)	–^d^
Age 5–17 years	0.0 (0/15)	0.0 (0/7)	0 (0 to 0)	–^d^
Charlson index score 0	4.5 (6/133)	0 (0/46)	−100 (−100 to −100)	–^d^
Charlson index score >0	0.0 (0/8)	0.0 (0/4)	0 (0 to 0)	–^d^
*Adult*	13.9 (51/367)	11.1 (37/333)	−23 (−51 to 22)	0.27
Age 18–64 years	10.5 (22/209)	3.9 (7/178)	−65 (−86 to −16)	0.02
Age 65+ years	18.3 (29/158)	19.4 (30/155)	7 (−39 to 88)	0.82
Charlson index score 0	9.2 (21/228)	12.6 (25/199)	42 (−23 to 262)	0.27
Charlson index score >0	21.6 (30/139)	9.0 (12/134)	−64 (−83 to −27)	0.005
Community acquired	11.2 (48/427)	9.5 (30/315)	−17 (−49 to 34)	0.45
Healthcare‐facility acquired	11.1 (8/72)	11.9 (7/59)	8 (−63 to 217)	0.89
Post‐neurosurgical	11.1 (1/9)	0.0 (0/9)	−100 (−100 to −100)	–^d^
Total	11.2 (57/508)	9.7 (37/383)	−15 (−45 to 31)	0.45

^a^ Plus–minus values are annual means ± standard deviations.

^b^ Per 100,000 person‐years.

^c^ The comparison of 5‐year mortality rates with logistic regression was performed after exclusion of cases that underwent any neurosurgical intervention during hospital in‐time.

^d^ Suppressed due to few observations.

### CBM and HBM

**Fig. 2:**
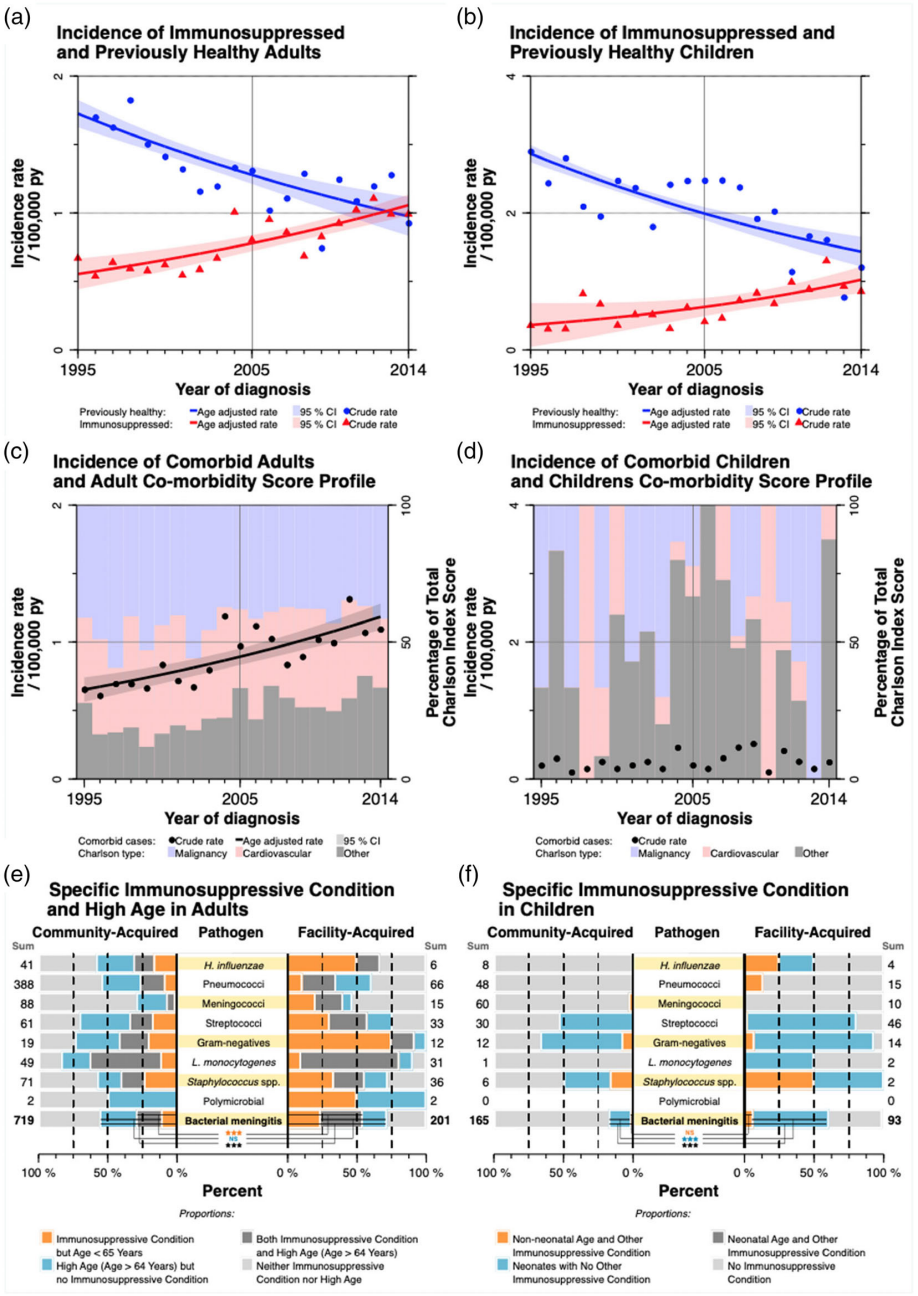
Incidence rate (IR) and immunosuppression of bacterial meningitis (BM). (a) The crude and predicted IRs for BM in adult patients with specific immunosuppression and without premorbid conditions at admission during the conjugate vaccine era, 1995–2014, are shown. Detailed definitions of premorbid conditions are available in the Supplementary Information (Materials and Methods, Tables S1 and S2). (b) The corresponding estimates are shown for children. (c) The crude and predicted IRs for BM in adult patients with Charlson comorbidities present at admission during the conjugate vaccine era on the left y‐axis (y1) and annual stacked proportions by patient Charlson comorbidity score during the conjugate vaccine era on the right y‐axis (y2). (d) The corresponding values for children. (e) The pathogen‐specific profiles of specific predisposing conditions and older age among adult community‐acquired episodes compared to adult (non‐neurosurgical) facility‐acquired episodes, post pneumococcal conjugate vaccine (PCV). (f) The pathogen‐specific profiles of individual predisposing conditions among children with community‐acquired episodes compared to those in children with (non‐neurosurgical) facility‐acquired episodes, post‐PCV. Post‐neurosurgical episodes are excluded from (e) and (f) as they are, by definition, preceded by a lesion resulting in dysfunction of the blood–brain barrier, a specific form of immunosuppression. CI95%, 95% percent confidence interval; H. influenzae, Haemophilus influenzae; L. monocytogenes, Listeria monocytogenes; Py, person‐years; Streptococci, non‐pneumococcal streptococci. Significance levels are shown as: ^*^p < 0.05; ^**^p < 0.01; ^***^p < 0.001; or nonsignificant. Predictions are depicted as median splines. The crude IR for adult patients without premorbid conditions in 1995 (IR = 2.07) is not shown in the graph.

CBM constituted 86% (9211/10,771) of the episodes (Table 1). Most of the episodes caused by *Haemophilus* (97%), meningococci (95%), pneumococci (90%), *Listeria* (69%) and streptococci (63%) were CBM. Pneumococci, *Haemophilus* and meningococci constituted 36%, 30% and 20%, respectively, of the CBM episodes (Table 1). The most common aetiology among CBM episodes post‐PCV in children was meningococcal infection (36%, 60/165), while pneumococci still dominated in adults (54%, 388/719) despite a reduced overall incidence of pneumococcal CBM episodes (−20%, CI95% −30 to −10, *p* = 0.001) (Fig. 2, Table 2, Fig. S1). HBM was observed in 1560/10,771 (14%) episodes (Table 1). Here, staphylococci dominated (30%), followed by pneumococci (24%) and streptococci (21%). Pneumococcal infection was the most common aetiology among HBMF episodes post‐PCV in both non‐neonatal children (36%, 15/42) and adults (33%, 66/201) (Table S5). HBMF episodes increased somewhat during the conjugate‐vaccine era (Figs S1 and S2). Among the associated clinical settings, nursing homes provided the highest proportion of *Listeria* (11%) (Table 1). Post‐neurosurgical BM was the least prevalent healthcare‐associated form (Table 1). Post‐PCV, the most common aetiology among post‐neurosurgical episodes in both children (61%, 14/23) and adults (44%, 53/121) was staphylococcal infection, but the incidence of pneumococcal post‐neurosurgical BM increased (106%, CI95% 2–319, *p* < 0.05) (Table S5, Table 2, Figs S1 and S2). Again post‐PCV, gram‐negative bacteria were more common among post‐neurosurgical episodes with an indwelling device present (23%, 9/39) than in those without (6%, 6/105) (Table S5).

### Premorbid conditions in patients with BM

Premorbid conditions were found in 20% (2157/10,771) of all the BM episodes (Table 1). The most common immunosuppressive premorbid conditions, in descending order, were traumatic, cerebrovascular and neoplastic lesions of the central nervous system (CNS) in 30% (652/2157), malignant neoplasia in 30% (641/2157) and rheumatic disease in 11% (241/2157). Premorbid conditions were most frequently found in patients infected with *Listeria* (58%), staphylococci (53%), gram‐negative bacteria (45%), multiple microbes (38%), non‐pneumococcal streptococci (24%) and pneumococci (24%) (Table 1). Following the introduction of the Hib vaccine, the incidence of BM patients with comorbidities increased (annual change 3%, CI95% 3–4, *p* < 0.001); at the end of the study period, there were more adult BM patients with immunosuppression than without previous morbidities (Fig. 2). Among the episodes caused by pneumococci, the most common premorbid condition was malignant neoplasia 31% (277/889). Cerebrovascular lesions and basilar skull fractures constituted 9% (81/889) and 7% (65/889), respectively, of pneumococcal episodes (Table 1). Malignant neoplasia in adults was most common in CBM caused by *Listeria*, followed by that caused by gram‐negative bacteria; the corresponding pathogens for children were staphylococci, followed by gram‐negative bacteria (Fig. S4). Adult patients infected with *Listeria* had the highest proportions of older age group individuals, rheumatic disease, and malignant neoplasia, while cerebrovascular lesions were most prevalent among adult patients with gram‐negative meningitis (Fig. S4). However, staphylococci were dominant in patients with a tumour or CNS haemorrhage (Table 1). Among the polymicrobial episodes, the most prevalent form of specific immunosuppression was malignant neoplasia 15% (6/40) (Table 1).

### Risk factors for acquiring RBM

In total, 6% (670/10,771) of BM episodes occurred in RBM patients and 80% of these (538/670) were first and second episodes. The majority were caused by pneumococci (57%, 381/670) and most occurred in the community (82%, 547/670) (Table S6). RBM was observed in 23% (9/40) of the episodes that occurred in adult patients with polymicrobial aetiology (Tables 1 and S6). After the introduction of ICD‐10 codes in 1997, primary immunodeficiencies and neurosurgical, traumatic, cerebrovascular and neoplastic lesions of the CNS were associated with RBM, and the recurrence rate increased over time (relative risk 1.1, CI95% 1.1–1.2, *p* < 0.001) (Tables 3 and S7).

**Table 3:** Risk factors for acquiring recurrent bacterial meningitis, Sweden 1997–2014. Multivariate survival model including age, gender and variables with a univariate p‐value <0.1 and a relative standard error <0.3 as estimated by using logistic regression with adjustment for calendar time and follow‐up time^a^. The adjusted relative risk for calendar time in 1‐year intervals (continuous variable) was 1.1 (1.1–1.2), p <0.001^b^. To further minimise potential bias due to different versions of the International Classification of Diseases (ICD), the analysis was restricted to the period after the introduction of ICD10 in the year 1997

Risk factors of recurrent bacterial meningitis	Adj. relative risk^c^ (95 % confidence interval)	*p*‐Value
**Age categories and gender**		
Age 0–4 years	(ref)	(ref)
Age 5–17 years	0.8 (0.3–1.8)	0.53
Age 18–64 years	0.9 (0.5–1.5)	0.67
Age 65 years or more	0.8 (0.4–1.4)	0.43
Male gender	1.1 (0.8–1.6)	0.59
**Lesions of the central nervous system** ^d^		
Intracranial shunt present	2.8 (1.4–5.5)	**0.002**
Cochlear implant present	8.0 (4.3–15.1)	**<0.001**
Basilar skull fracture	3.7 (1.6–8.3)	**0.002**
Meningeal tumour^e^	4.8 (1.4–16.2)	**0.01**
Ischemic cerebrovascular	2.9 (1.5–5.7)	**0.001**
**Malignant neoplasia**		
*Haematological malignancy*		
Lymphoma	2.4 (0.7–7.8)	0.16
Leukaemia	2.0 (0.6–6.9)	0.30
*Other malignant neoplasia*	1.3 (0.6–2.6)	0.46
**Comorbidity**		
Moderate to severe liver disease	2.8 (0.9–9.1)	0.09
**Primary immune deficiency**		
Humoral deficiencies	17.9 (6.5–49.4)	**<0.001**
Complement deficiencies	7.2 (1.6–32.8)	**0.01**
Other deficiencies	3.1 (0.3–35.8)	0.37
Monoclonal gammopathy of unknown significance	5.8 (1.8–18.6)	**0.003**

^a^ Only the first and second episode per patient were retained for this analysis.

^b^ Including all recurrent episodes, first time episodes excluded.

^c^ Relative risk estimated using negative binomial regression clustered on patients with the logarithm of time as an offset variable and robust variance estimation.

^d^ Neurosurgical, traumatic, neoplastic and cerebrovascular lesions of the central nervous system.

^e^ Including malignant and benign tumour forms.

### Risk factors for severe sequelae and the disease burden of BM

**Fig. 3:**
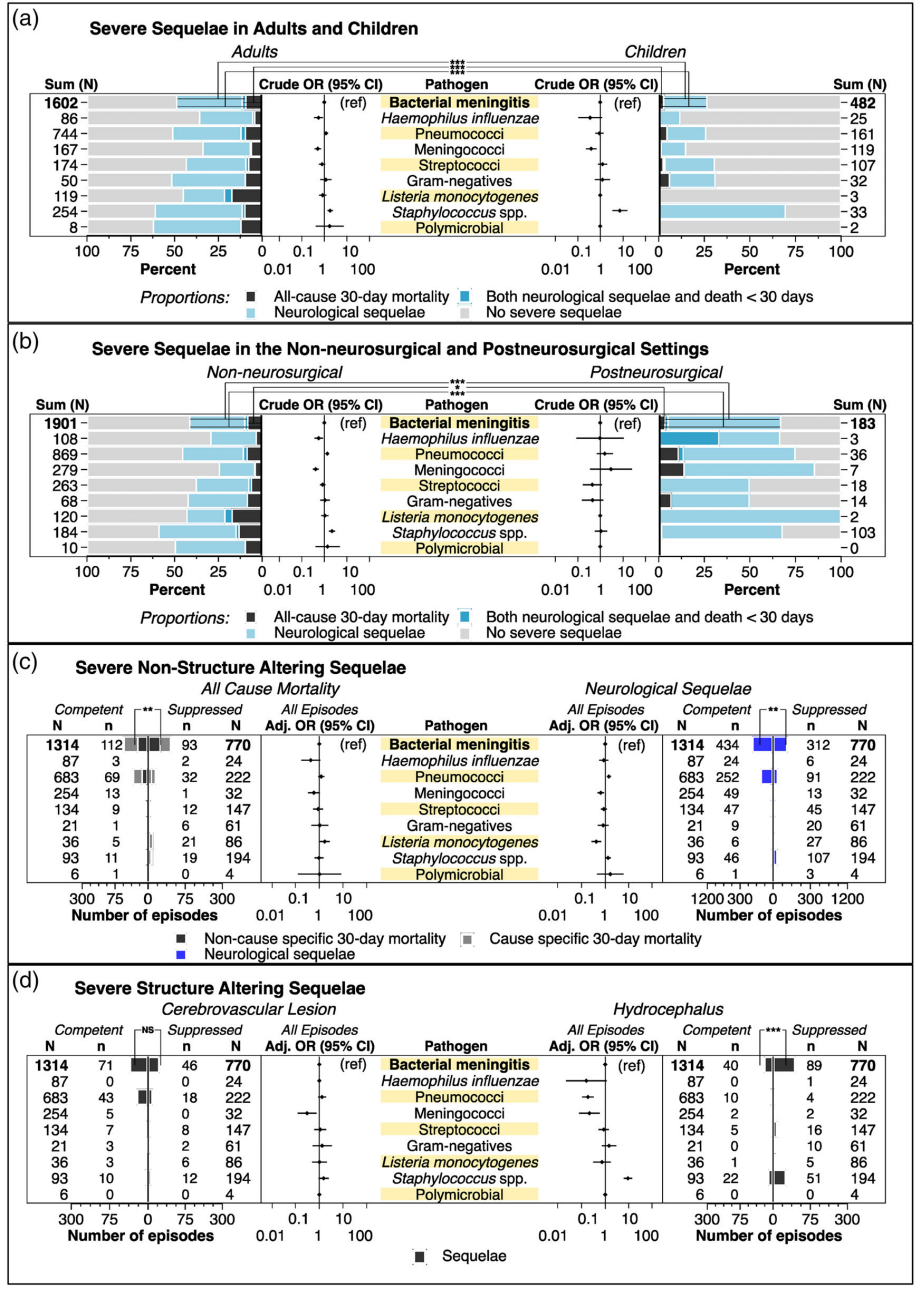
Severe sequelae (SS) of bacterial meningitis. (a) The unadjusted odds ratios (ORs) with 95% confidence intervals (CI95%) of meningitis‐causing pathogens and the univariate analysis of age and SS, consisting of all‐cause 30‐day mortality and severe neurological sequelae (including structural and nonstructural forms). The OR for age and SS was 2.6 (CI95% 2.1–3.3, p < 0.001), for age and all‐cause 30‐day mortality was 3.9 (CI95% 2.3–6.6, p < 0.001) and for age and severe neurological sequelae was 2.1 (CI95% 1.7–2.7, p < 0.001). (b) The corresponding distributions and analysis of SS stratified by clinical setting. The OR for clinical setting and SS was 2.9 (CI95% 2.1–3.9, p < 0.001), for clinical setting and all‐cause 30‐day mortality was 0.5 (CI95% 0.3–1.0, p = 0.04) and for clinical setting and severe neurological sequelae was 3.5 (CI95% 2.5–4.8, p < 0.001). (c) The univariate analysis of immunocompetence and all‐cause 30‐day mortality was 1.5 (CI95% 1.1–2.0, p = 0.009), and the adjusted ORs for pathogen‐specific and all‐cause 30‐day mortality are shown on the left. The proportion of the all‐cause mortality that was cause specific is also shown. On the right, the corresponding analyses of immunocompetence, pathogens and nonstructural neurological sequelae are shown. (d) The corresponding stratified distributions and analysis for structural neurological sequelae. The OR for immunosuppression and hydrocephalus was 4.2 (CI95% 2.8–6.1, p < 0.001). Logistic regression was used for univariate and multivariate analysis; the multivariate analysis included adjustment for sex, age and clinical setting. Significance levels are shown as: ^*^p < 0.05; ^**^p < 0.01; ^***^p < 0.001; or nonsignificant. Pharmacological data were available from 2005 onwards; data on SS were therefore restricted to episodes reported thereafter. Adj., adjusted; Ref, reference category; Streptococci, non‐pneumococcal streptococci.

After 2004, the overall rate of severe sequelae following single‐episode BM was 44% (917/2084), and patients on average lost 11 years of healthy life due to BM (Tables S8 and S9). The risk of severe sequelae was higher among adults (49%, 788/1602) than children (27%, 129/482) (OR 2.6, CI95% 2.1–3.3, *p* < 0.001), and higher in the post‐neurosurgical setting (67%, 123/183) than in the non‐neurosurgical setting (42%, 794/1901) (OR 2.9, CI95% 2.1–3.9, *p* < 0.001) (Fig. 3, Table S8). The highest severe sequelae rates were found among patients with staphylococcal (62%, 179/287), polymicrobial (50%, 5/10) and pneumococcal (47%, 425/905) infections (Table S8). The rate of neurological sequelae was 36% (746/2084), with a higher rate in adults (39%, 632/1602) than in children (24%, 114/482) (OR 2.1, CI95% 1.7–2.7, *p* < 0.001), and higher in the post‐neurosurgical setting (63%, 116/183) than in the non‐neurosurgical setting (33%, 630/1901) (OR 3.5, CI95% 2.5–4.8, *p* < 0.001) (Fig. 3, Table S8). The rate of nonpsychological neurological sequelae was 28% (588/2084) and the rate of psychological sequelae was 13% (261/2084). The rate of nonstructural neurological sequelae was 29% (607/2084) as compared to 11% (232/2084) for structure‐altering neurological sequelae, most commonly hydrocephalus (6%, 129/2084), followed by cerebrovascular lesions (6%, 117/2084) (Fig. S5). Hydrocephalus was more common in episodes caused by staphylococci (OR 9.2, CI95% 6.1–13.9, *p* < 0.001) (Fig. 3). In the non‐neurosurgical setting, there was no association between HBMF and severe sequelae (OR 1.0, CI95% 0.8–1.3, *p* = 0.76) (Table S10).

### All‐cause 30‐day mortality due to BM

The overall 30‐day mortality of BM was 9% (981/10,771); it was 3% in children and 14% in adults, with a decreasing trend since 1987 in adults, particularly among the elderly (annual change −2%, CI95% −4 to −1, *p* < 0.001), but also among nonvaccinated adults (annual change −3%, CI95% −5 to −2, *p* < 0.001) (Fig. S6). The same trend was observed for pneumococcal BM, with a decreasing trend in mortality overall (annual change −3%, CI95% −5 to −2, *p* < 0.001) in the CBM setting (annual change −3%, CI95% −5 to −2, *p* < 0.001) and in adults since 1987, especially among the elderly (annual change −4%, CI95% −6 to −2, *p* < 0.001) (Figs S6 and S7). When comparing the pneumococcal episodes from 5 years before and after PCV introduction, mortality was reduced in children (no deaths post‐PCV) and in adults with comorbidities (−64%, −83 to −27, *p* = 0.005), as well as in adults ≤65 years (−65%, −86 to −16, *p* = 0.02) (Table 2). From the year 2005 onwards, the overall 30‐day mortality of BM was 10% (205/2084), with a higher rate in adults (12%, 189/1602) than in children (3%, 16/482) (OR 3.9, CI95% 2.3–6.6, *p* < 0.001) (Fig. 3). The mortality rate was higher among immunosuppressed patients (OR 1.5, CI95%, 1.1–2.0, *p* = 0.009), and one third of all fatalities in children were neonates (Table S8, Fig. 3). In the non‐neurosurgical setting, there was an association between older age (≥65 years) and mortality (adjusted OR 8.7, CI95% 3.7–20.3, *p* < 0.001) (Table S10).

## Discussion

BM is a devastating disease carrying a risk of death and severe neurological sequelae among survivors. To develop effective treatment and preventive strategies, we need knowledge on incidence trends, aetiology and risk factors over the long term. In this unique national population‐based study, we examined BM over 50 years in Sweden using national registries. We found that the incidence of BM decreased dramatically in young children after the introduction of conjugated vaccines into the childhood vaccination program, leading to changes in the aetiology of BM during the study period, which varied with age group. The most dramatic decrease was observed following the introduction of the Hib vaccine in 1993, in accordance with previous studies [15]. The incidence of Hib has remained low since then, probably because nonvaccine‐type strains have not expanded to any great extent in healthy carriers [16]. The incidence of pneumococcal BM also decreased after PCV introduction but not in nonvaccinated adults, which is in agreement with earlier studies showing a limited impact of PCVs on invasive pneumococcal disease in this age group [17, 18]. This limited effect is probably influenced by the observed replacement in carriage during childhood of vaccine‐type strains with nonvaccine type strains, which may spread to susceptible individuals, such as nonvaccinated adults [19]. Nonvaccine types have, in previous studies, appeared less capable of causing invasive diseases, including BM, in previously healthy individuals; they primarily affect the elderly and immunocompromised individuals, and generate a milder disease course [18, 19, 20]. We found that the incidence of meningococcal meningitis has decreased since the mid‐1970s, particularly in young children, even though a general meningococcal vaccination was not introduced. The same trend has been observed in other European countries, including Finland, and several mechanisms have been proposed [21]. The incidences of neonatal BM and BM caused by pathogens associated with post‐neurosurgical BM, *Staphylococcus* spp. and gram‐negative bacteria were in accordance with those found in previous studies [22, 23].

We also showed that during the conjugate‐vaccine era, the incidence of BM in patients with immunosuppression surpassed that of BM in previously healthy individuals. At the same time, the proportion of pathogens associated with immunosuppression (*Listeria monocytogenes*, *Staphylococcus* spp., gram‐negative bacteria) and RBM increased. These trends are probably the result of demographic changes, including increased age‐specific survival among adults [7, 8, 9]. In line with previous findings, we found that several neoplastic, cerebrovascular and traumatic CNS lesions associated with blood–brain barrier dysfunction are risk factors for acquiring BM [3, 24, 25, 26, 27, 28, 29, 30].

Importantly, the overall mortality was 9%, and this decreased during the study period (1987–2014) in the elderly and unvaccinated adults, while it remained low in children (3%). At the beginning of the study period (1987–1991), the all‐cause 30‐day mortality among adult patients with BM was 16% (126/780); towards the end of the study period (2010–2014), it was 9% (85/910) (OR 0.5, CI95% 0.4–0.7; *p* < 0.001). Mortality due to pneumococcal BM decreased during the study period, from 18% (94/524) at the beginning of the study period to 9% (42/466) by the end of the study period (OR 0.5, CI95% 0.3–0.7; *p* < 0.001). Post‐PCV, a decrease was observed among children, comorbid adults and the elderly. Moreover, severe sequelae were present in 44% of the patients with BM, and the highest rates were found among patients with staphylococcal (62%) and pneumococcal (47%) infections. Thus, despite decreased incidence and improved prognosis, we found that BM patients, on average, lost about 11 years of healthy life, and pneumococcal BM takes the highest toll. These data show similar patterns to those seen in previous studies, but with slightly higher rates [21, 31]. Furthermore, we showed that age, pathogen and immunocompetence, including prior neurosurgery, are important prognostic indicators for BM. However, in a non‐neurosurgical setting, patient age, rather than premorbid conditions or recent association with a healthcare facility, is of prognostic value. When considering initial empiric treatment in a patient with suspected BM, factors associated with opportunistic or resistant bacteria, such as immunosuppression, older age or recent hospital or nursing home care, are also important.

This study has several strengths but its limitations should also be considered. The study period was long, at 50 years; the source has national coverage and includes culture‐negative episodes. Random erroneous reporting would result only in nondifferential misclassification, and the comorbidity data have been shown to be highly valid [32]. The Swedish setting, with a tax‐financed healthcare system and unique individual numbers assigned to citizens at birth and to immigrants at contact with the healthcare system, allows for data tracking. NPR coverage has increased gradually from only a few hospitals in 1965 to near national coverage in 1975; thus, data from prior to 1975 should be interpreted with caution. We found no evidence of changes in data quality from 1987 onwards; thus, only data from this period were retained for the analysis. The medical records of 46 consecutive episodes of pneumococcal BM with 1 year of follow‐up data from hospital admittance were retrieved from selected Swedish secondary and tertiary care hospitals from 2005 to 2014, and we found consistency between the reported registry data and original medical records (Supplementary Information, Materials and Methods, Fig. S2).

We conclude that this national study provides important information on BM and its consequences, with a decreasing incidence in younger age groups in the post‐vaccination era, but with changes in aetiology and patient characteristics. Conjugated vaccines have reduced the number of BM cases caused by pneumococci and *H. influenzae*, but BM caused by meningococci has also decreased, even though no vaccine has been introduced against this pathogen. This information is relevant for future strategies to treat and prevent this severe infection.

## Author contributions

Nils Block, Pontus Naucler and Birgitta Henriques‐Normark designed the study. Nils Block, Pontus Naucler, Birgitta Henriques‐Normark and Philippe Wagner analysed the data. Statistical analyses were performed by Nils Block and checked by Philippe Wagner. Nils Block, Pontus Naucler and Birgitta Henriques‐Normark wrote the manuscript, with contributions from the other authors. All authors have read and approved the final version of the manuscript.

## Conflict of interests

The authors declare that no competing interests exist.

## Supporting information

Supplementary Appendix: Material and Methods
