# Supplementary material for: Bacterial meningitis: Aetiology, risk factors, disease trends and severe sequelae during 50 years in Sweden
Source: J Intern Med. 2022 Apr 3;292(2):350–64. doi: 10.1111/joim.13488 (PMC9544249; doi:10.1111/joim.13488)
Supplement: Supplementary file 1 — Supplementary Appendix: Material and Methods [file JOIM-292-350-s001.pdf]

SUPPLEMENTARY APPENDIX

SUPPLEMENTARY MATERIAL and METHODS

Age Groups and Premorbid Conditions

Unless otherwise specified, co–morbidities, risk factors of bacterial meningitis and pharmacological immunosuppression were defined as having information within five years before admission of:

- *co-morbidity*, defined as constituents of the Charlson co-morbidity index
- *risk factors of bacterial meningitis*, defined as primary immune deficiency, basilar and compound cranial fractures (information collected from the start of the study in 1964) including facial bones (except the mandibular bone)
- *pharmacological immunosuppression* defined as systemic steroid treatment (ongoing for at least two weeks and reported within one year of admission)

"Specific immunosuppressive conditions was defined as both specific Charlson co-morbidities and other immunosuppressive conditions: malignant neoplasia, rheumatic disease, diabetes with end organ damage, renal failure, pharmacological immunosuppression, patients of neonatal age, primary immune deficiency and neurosurgical, premorbid traumatic* (basilar and compound cranial fractures and parameningeal bleeds) and non-traumatic (ischaemic and haemorrhagic cerebrovascular lesions, benign and malignant neoplastic lesions including tumours of the brain parenchyma, spinal medulla and the enclosing meninges) lesions of the CNS resulting in blood-brain barrier dysfunction(1-8).

* Hospital classification codes used to identify a premorbid traumatic lesion of the central nervous system

**Traumatic lesion**	**ICD10**	**ICD9**	**ICD8**
Traumatic para-meningeal bleed	S064+01	852+AB	852,00 10 90
	S065+01		
	S066+01		
Compound (open) fracture	S0271	800+CD	800,10
	S0281	802+FX	802,52
	S0291	803+CD	803,10
		804+CD	804,10
	S0221	802B	802,10
	S0231	802H	
	S0241	802F	
Basilar skull fracture	S021+01	801+A–D	

Age was presented as standard age groups; neonates (admitted at age < 29 days postpartum), children (young children 0–4 and 5–17 years of age) and adults (18–64 and the elderly over 65 years of age).

Healthcare-associated Bacterial Meningitis

Healthcare-associated bacterial meningitis (HBM) was defined in accordance with Durand et al and the Centers for disease control and prevention/National Healthcare Safety Network surveillance criteria from the year 2017 for healthcare-associated infection and meningitis following neurosurgery within 90 days and/or the presence of an indwelling device. The

included surgical procedures together with their hospital codes are specified below. In Sweden, the K6-classification of surgical procedures was used during the years 1963 to 1996 and the KVÅ-classification of surgical procedures was used since the year 1997.

Episodes admitted with a meningitis diagnosis within 90 days of neurosurgery (ICD10-8; T850 T814 998F DA016 JAL50–51 DD006 DD010 DD018 DFE00 GA003 DA002 DA004 998,50 998,45, KVÅ; AA|A–H J-N P U W AB|A–E W AEA AW|A–E W, K6; 01|0–1 3-5 8–9 02|0–4 03|0–5 8–9 2058 06|18–19) without the placement of an indwelling intracranial device and cases related to an indwelling device (***device-related bacterial meningitis*****,** ***DBM)*** including an intracranial shunt (K6:02|20 22 and KVÅ:AAF|05 10 15 40), electrode (K6:02|40 41 42 and KVÅ:AAG20 AAW01 AEA00) or a cochlear implant (K6:2058 and KVÅ:DD|006 010 018 DFE00 GA003) present on admission were classified as ***post-neurosurgical bacterial meningitis (PBM)***

Neonatal episodes, that did not fulfill the criteria of PBM, were defined as ***facility-acquired bacterial meningitis (HBMF)*** at age > 48 h if caused by pathogens other than non-pneumococcal streptococci and at age > 6 days if caused by non-pneumococcal streptococci. For non–neonatal patients that did not fulfill the criteria of PBM, we used bacterial meningitis specific temporal cut-offs for *HBMF*-episodes at > 48 h of admission and < 7 days of discharge.

In addition to a focus of infection** present or incubating at admission, we used a secondary attribution period of 14 days before admission due to bacterial meningitis to differentiate episodes secondary to a community-acquired primary focus of infection from *HBM*-episodes. The difficulty of establishing a route of infection in episodes with penetrating head-trauma followed by neurosurgery has been recognized previously. Therefore, BM-episodes where no temporal relation between BM-onset, trauma and neurosurgery could be established were classified as post-neurosurgical.

** Hospital classification codes used to identify a focus of infection present or incubating at admission:

**Infection focus**	**ICD10**	**ICD9**	**ICD8**
Infective endocarditis	I330	421A	421,00
		036E	
Ear focus	H660	382A	381,00
	H700	383A	382,00
Sinusitis	J010-4	461A-D|WX	461,00-04|09
	J019		
Tonsillitis	J030	034A	034,00
	J369	475	501,99
Bacterial lung infections	J139	481	481,99
	J149	482C-E	482,10-30
	J152-4	507A	485,01
	J690	510|AX	510,01|03|09
	J851	513A	513,99
	J860		
	J869		
Abscess of the CNS	G060	324ABX	322,00|03
	G061|AJKX		

Analysis of ICD-coding practices over time

To assess changes in the sensitivity of ICD-coding practices over time, we analysed the annual incidence rate of acute cystitis and pyelonephritis in non-pregnant and non-puerperal women and males between 18 and 40 years of age hospitalized with bacterial meningitis without a specified aetiology, meningitis/encephalitis with herpes simplex or meningitis/encephalitis with varicella zoster between in Sweden between 1987 and 2014. This separate cohort was used since the incidence rate of acute cystitis and pyelonephritis over time was expected to be constant. Therefore, any temporal trends in incidence rate over time would suggest changes in coding practices over time. We found no evidence of significant changes in sensitivity of the analysed ICD-codes over time:

**Time period in years**	**n/N:**	**P-value for annual change#**
1987 to 2014 (ICD9 and ICD10 used subsequently)	40/1219	0.106

#Negative binomial regression was used to analyse changes in incidence rate over time

Recurrent Bacterial Meningitis

Episodes that required less than three days (unless death occurred within that time) of hospitalization were excluded to minimize the risk of misclassification. We used a washout-time consisting of the most common recommended therapy-duration (10 days) in Sweden and an additional three weeks in accordance with prior definitions. In addition, new episodes registered less than 30 days from hospital discharge date were excluded to account for late occurring SS relating to the index-episode and to ensure a complete convalescence period between episodes in the same patient

Severe sequelae

Severe sequelae were defined as all-cause mortality within thirty days and severe neurological sequelae within one year (cerebrovascular sequelae restricted to 90 days) after admission that were not present prior to the current hospital admission due to bacterial meningitis:

All-cause mortality

Deaths < 30 days specified as attributable to bacterial meningitis by a physician (Cause specific mortality)

Deaths < 30 days not specificed as attributable to bacterial meningitis by a physician (Non-cause specific mortality)

Severe neurological sequelae

Structure altering ("Structural") lesions

- Haemorrhagic and ischaemic cerebrovascular lesion
- Hydrocephalus

Neurological symtoms

- Epilepsy
- Paresis of one or more limbs
- Loss of vision and/or other cranial nerve dysfunctions excluding hearing impairment

Hearing loss

- Sensorineural hearing impairment

Psychological symtoms

- Depression and anti-depressive pharmacological treatment *(starting July 2005)*
- Moderate and severe anxiety *(starting July 2005)*
- Attention deficit hyperactivity disorder (ADHD)

Disease Burden

Cases with information on severe neurological sequelae were assigned standard disability weights per the Global Burden of Disease (GBD) 2013 to account for multiple SS. Years lived with disability (YLD) and years of life lost (YLL) were calculated using life span or data on age and gender-specific life expectancy at year of diagnosis from Statistics Sweden. Disease burden constituents were defined in accordance with the GBD as episodes with acute consequences and SS.

Formula for calculation of **disability adjusted life years (DALY)** constituents:

Years lived with disability (YLD): n cases x duration until remission or death x DW (disease weight)

Years of life lost (YLL): n cases x (national) life expectancy at time of death

Disease weights (DW) and ICD-codes:

Episodes

- *Acute episode, severe*: 0.133
- *Post-acute effects*: 0.219

Severe neurological sequelae

- *Severe structure-altering neurological sequelae*

Stroke, long term consequences, moderate + cognition problems:

0.316*life expectancy at time of admission

ICD8-10: 321,00 09 430,00-1 08-09 90-91 98-99 431,00-01 08-09 90-91 98-99 432,00-02 08 09 90-92 98-99 433,00 99, 434,00 99 436,00 99 437,00 99 | 325 430 431 432ABX 433+ABCDWX, 434+ABX 437G 671F | G089 I601-3 I610-16 I618-21 I629 I630-36 I638-39 I649 I676 Z867C

Hydrocephalus (moderate motor + cognitive impairment): 0.203* life expectancy at time of admission ICD8-10: 324,00, 347,93 | 331DE | G910-12, G918+ABW, G919

- *Severe non-structure-altering neurological sequelae*

Epilepsy, severe followed by less severe for the remaining life expectancy

First year: (0.552*1) + (0.263* life expectancy at time of admission-1)

ICD8-10: 345,20 345,00 09-11 19 30-33 39 98 99 | 345, 345JKLMNPQWX | G400+ABW G401ABCDEX G402+ABCDEFX G403+ABCDEFX G404+ABCDX G405-9 G418-19

Motor, severe (see below, hemi/paraplegia)

0.402*life expectancy at time of admission

ICD8-10: 344,00-03 08 09, 342+ABX, 344ABCDEFGWX, G810, G811, G819-25, G830-34, G838-39

Motor, moderate (see below, ataxia/coordination)

0.203* life expectancy at time of admission

ICD8-10: 780,40 49 | 781CD | R260-3, R268, R270, R278

Blindness (see below, uni/bilateral)

0.187*life expectancy at time of admission

ICD 8-10: 379,09/19/29/39 | 369ABG | H488, H740-7, H532-4, H541-2, H559, H540+H544

Severe vision impairment, (see below, bilateral vision loss)

0.184*life expectancy at time of admission

ICD 8-10: 781,00/01/02/10/12 377,90 | 368CDE, 369CD, 377BDEWX, 379F | H470-77, H488, H532-34, H541-42, H559

Moderate vision impairment, (see below, unilateral vision loss)

0.031*life expectancy at time of admission

ICD 8-10: 781,09 | 369H-X | H545-H546, H549

Mild vision impairment, (see below)

0.003*life expectancy at time of admission

ICD 8-10: 367,00-03+09, 373,00-04+09 | 036W, 368B, 377D, 378A-X | H469, H481, H490-94, H490-506, H508-512, H518-19, H535-36, H538-39

Other cranial nerve dysfunctions (stroke, long term consequences, moderate, see below)

0.070*life expectancy at time of admission

ICD 8-10: 356,00-05, 356,8-09, 781,50-52, 781,59, 781,60-61, 781,63-64, 781,66 | 350BWX, 351AWX, 352ABCDEFGX, 781B, 782A, 784DFG | G500-001, G508, G510-514, G518-23, G527-29, G531, R200-203, R430-32, R438, R470-71, R478, R480-82, R488

Severe depression

0.658*life expectancy at time of admission
ICD10: F322-23

Moderate depression
0.396*life expectancy at time of admission
ICD10: F321

Mild depression
0.145*life expectancy at time of admission
ICD10: F320, F328-29, F412

Severe anxiety
0.523*life expectancy at time of admission
ICD10: F410-11

Moderate anxiety
0.133*life expectancy at time of admission
ICD10: F400, F412-13, F418-19, F930-32

Attention deficit hyperactivity disorder (ADHD)
0.045* life expectancy at time of admission
ICD10: F900+ABCX

Hearing loss (assumed severe for all expect those with information of a hearing aid which are classified as mild, see below) 0.158*life expectancy at time of admission
ICD10: H903-8, H912-13, H918-19

With hearing aid: 0.010*life expectancy at time of admission
ICD10: Z461, AD051, DEE00, DDE00, DFE10, GA004, GA005, GA022

SUPPLEMENTARY TABLES

Table S1: Meningitis-causing bacteria according to Swedish ICD 8, 9 and 10

**Pathogen**	**ICD10**	**ICD9**	**ICD8**
H. influenzae	G000	320A	320,00
S. pneumoniae	G001	320B	320,10
N. meningitidis	A390	036A	036,00
Non-pneumococcal streptococci	G002	320C	320,80
Gram-negatives<br>(except *H. influenzae* and<br>*N. meningitidis*)	G008A<br>G008B<br>G008W & A022<br>G008W & A010<br>G019 & A022<br>G019 & A010<br>G008W & B965<br>G019 & B965<br>G008W & B964<br>G019 & B964<br>G008W & B966<br>G019 & B966<br>G008W & B968B<br>G019 & B968B	320W & 041E<br>320W & 041D<br>320W & 003C<br>320W & 002A<br>320H & 002A<br>320W & 041H<br>320H & 041H<br><br>320W & 041G<br>320H & 041G	320,88 & 038,80<br>320,99 & 038,80
L. monocytogenes	A321	320H & 027A	027,01
Staphylococcus spp.	G003	320D	320,88 & 038,10<br>320,99 & 038,10

Table S2. Charlson co-morbidities and primary immunodeficiency according to Swedish ICD 8, 9 and 10

A. VASCULAR CO-MORBIDITIES

Acute coronary syndrome	ICD 10	ICD 9	ICD 8
Unstable angina pectoris	I200	411B	411,00 ,99**
Myocardial infarction (MI)	I21-22	410+ABWX	410,00 ,99
Post MI syndrome	I241	411C	-
Previous MI	I252	412	412,01 91

*Congestive heart failure**	ICD 10	ICD 9	ICD 8
CHF	I50	428+ABX	427,00 428,99
HT disease w CHF	I110 I130 I132	-	-
Pulmonary heart disease	I279	416+ABX	426,01 02 08 09
CHF of the neonate	P290	-	-

* excluding arrhythmia and valvular disorders

*Peripheral vascular disease**	ICD 10	ICD 9	ICD 8
Atherosclerosis	I70	440+A-CWX	440,00 10 20 38 99
Aortic aneurysm/dissection	I71	441+A-H	441,00 10 20 99
Non-aortic aneur./dissec.	I72	442+A-DWX	442,00 10 20 99
Other periph. vasc. disease	I739B	443X	443,90

* excluding hypertension

Cerebrovascular disease	ICD 10	ICD 9	ICD 8
Haemorrhagic cerebrovascular lesion (CVL)	I60-62	430-431<br>432+ABX	430-431,00 01 08 09 90 91 98 99
Haemorrhagic CVL (neo.)	P52	772B+C	-
Perinatal haemorrh. CVL (neo.)	P10	767A	772,01-03 778,20
Ischaemic CVL	I63	433+A-DWX<br>434+ABX	432,00 01 02 08 09 90 91 92 98 99<br>433-434,00 99
Cerebrovascular disease (CVD)	I67<br>I649 (CVL NOS)<br>G459 (TIA)	437+A-GWX<br>436 (CVL NOS)<br>435 (TIA)	437-438,00 99<br>436,00 99 (CVL NOS)<br>435,00 ,99 (TIA)
Late effects of CVD	I69	438	-
Trombophleb. of venous sini	G089	325	321,00 09
Cerebr. ven. thromb. (preg.)	O225	671F	674,99 (all CVD)
Cerebr. ven. thromb. (puer.)	O873	674A	674,99 (all CVD)

B. PEPTIC ULCER

	ICD 10	ICD 9	ICD 8
	K25-27	531-34	531-34

C. DEMENTIA

	ICD 10	ICD 9	ICD 8
	F00-03, G30, G318A	290 291C 294B 331A-C (+F-H)	290 292,00

D. CHRONIC PULMONARY DISEASE

	ICD 10	ICD 9	ICD 8
Emphysema	J43	492	492
Chronic obstructive lung disease	J44	496	491,04
Asthma (excluding episodes specified as acute)	J45 0189	493	493
Bronchiectasis	J479	494	518
Pneumoconiosis	J60-65	500-503, 505	515
Chronic lung disease due to chemicals, gas, smoke or vapour	J684	506E	516
Interstitial lung disease	J703, J84	515-516	517
Primary pulmonary hypertension	I270	416A	426,02
Cystic fibrosis	E84	277A	273,00
Congenital conditions of the lung	Q33	748 EFGW	748
Chronic perinatal respiratory disorders	P27	770H	776,60 70

E. RHEUMATIC DISEASE AND ASSOCIATED CHRONIC AUTOIMMUNE CONDITIONS (PSORIASIS, INFLAMMATORY BOWEL DISEASE AND PYODERMA GANGRAENOSUM)

	ICD 10	ICD 9	ICD 8
Rheumatoid arthritis	M05-06	714	712
Psoriasis	M07 L40	694D 696AB	696, 00-29+98
Juvenile arthritis	M08-09	(714D, above)	(712,00, above)
Vasculitis and polyarthritis	M30-31 D891	446 273C	446 275,40
Systemic lupus erythematosus	M32	710A 695E	734,10 695,40
Polymyositis and dermatopolymyositis	M33	710DE	716
Systemic scleroderma	M34	710B	734,00-09
Other autoimmune systemic disorders	M35	710CWX	734,90-99
Morbus Bechterew	M45	720A	712,40
Pyoderma gangraenosum	L889	-	-
Morbus Crohn	K50	555	563,00
Ulcerous colitis	K51 0234589	556	563,10

F. MILD LIVER DISEASE

Without indication of cirrhosis/fibrosis/failure/ infarction/coma or varices of the oesophagus	ICD 10	ICD 9	ICD 8
Viral hepatitis	B16 19 B17 0	070 DFX	070,02-03 999,20

	B18 0+A-D+HWX<br>B18 1+A-D+HWX<br>B18 2+A-D+HWX<br>B18 8<br>B18 9+A-D+HWX<br>B199		
Liver disease related to the consumption of ethanol	K70 019	571ABD	-
Toxic liver disease	K71 02345689	573D	-
Chronic hepatitis NOS	K73	571E	-
Other inflammatory hepatitis and abscess of the liver	K75 048	572AB	572,99
Other conditions of the liver	K76 0189<br>K77<br>Q446	571 WX<br>573ABCWX	573,01

G. DIABETES MELLITUS*

Without indication of end organ damage	ICD 10	ICD 9	ICD 8
Type 1, no complications indicated	E109 E106A	250A	250,00 09
type 1 with coma	E100	250C	250,07
type 1 with ketoacidosis	E101	250B	-
Type 2, no complications indicated	E119 E116A	- (type not specified in ICD 9)	- (type not specified in ICD 8)
type 2 with coma	E110	-	-
type 2 with ketoacidosis	E111	-	-

* Not including codes for diabetes mellitus during pregnancy

H. DIABETES MELLITUS WITH END ORGAN DAMAGE

With indication of end organ damage	ICD 10	ICD 9	ICD 8
Type 1, with complications	E102-8 (except E106A)	250 D-X<br>357C	250,01-06 08
Type 2, with complications	E112-8 (except E116A)<br><br>H280 H360<br>G590 G632<br>M142<br>M908FGH<br>N083	- (type not specified in ICD 9)	- (type not specified in ICD 8)

I. HEMI AND PARAPLEGIA

	ICD 10	ICD 9	ICD 8
Hemi- and paraplegia	G81-82	342-43<br>344AB	343,00 02<br>344,00-01

J. MODERATE OR SEVERE RENAL DISEASE

	ICD 10	ICD 9	ICD 8
	N18 3-9	585<br>403-4	582<br>792

K. MODERATE OR SEVERE LIVER DISEASE

With indication of cirrhosis/fibrosis/failure/ infarction/coma or varices of the oesophagus	ICD 10	ICD 9	ICD 8
Viral hepatitis	B150<br>B16 02<br>B18 0EFG<br>B18 1EFG<br>B18 2EFG<br>B18 9EFG<br>B190	070 ACEG	-
Liver disease related to the consumption of ethanol	K70 234	571C	571,00-01
Toxic liver disease	K71 17	-	-
Liver failure	K72	-	-
Fibrosis, cirrhosis and sclerosis of the liver	K74	571 FG	571,90-99
Thrombosis of liver veins	I819 I820 I823	452 453A	452,99
Other conditions of the liver and signs of liver failure	K76 234567<br>I98 23	572CDEW<br>573E<br>456ABC	573,02<br>456,00

L. LYMPHOMA AND PRIMARY IMMUNE DEFICIENCY; MONOCLONAL GAMMOPATHY OF UNKNOWN SIGNIFICANCE (MGUS)

	ICD 10	ICD 9	ICD 8
Lymphoma	C81-86	200-202	200-202
MGUS	D472	273B	-

M. LEUKEMIA, MALIGN PLASMA CELL NEOPLASIA AND ASSOCIATED CONDITIONS OF IMMUNOSUPPRESSION (MYELOFIBROSIS, MYELODYSPLASTIC SYNDROME AND PRIMARY IMMUNE DEFICIENCIES EXCLUDING MGUS)

	ICD 10	ICD 9	ICD 8
Leukaemia	C91-95	204-208	204-207
Myelodysplastic syndrome	D46 679	-	-
Myelofibrosis	D474	289W	209,99
Malign plasma cell neoplasia	C90	203 238G	203

(excluding lymphoplasmacytic lymphoma and MGUS)			
Humoral deficiencies	D80 (excl. D802)	279J	270 00 10
Severe combined immunodeficiency (SCID)	D81	(not specified in ICD 9)	(not specified in ICD 8)
Common variable immunodeficiency (CVID)	D83	(not specified in ICD 9)	(not specified in ICD 8)
Complement deficiencies	D841	279W	(not specified in ICD 8)
Other primary immune deficiencies	D82 (excl. D824AB)<br>D84 0 89<br>G113	279LMX<br>334W	759,86

N. METASTATIC SOLID TUMOUR

	ICD 10	ICD 9	ICD 8
	C77-78<br>C79<br>0123*456789	196-97<br>198ABCD*EFGHW	196-97<br>198 01 18 21 31*<br>48 51 98

* Metastasis to the central nervous system (CNS) is scored as 6 points when calculating the Charlson co-morbidity index but categorized as a lesion of the CNS

O. ANY (OTHER) TUMOUR

	ICD 10	ICD 9	ICD 8
Malign tumours, excluding the central nervous system (CNS) and the meninges	C00-69<br>C72 12345<br>C73-75<br>C97	140-190<br>192AWX<br>193-95	140-190<br>192 10 21 31<br>193-95
Benign or malign brain parenchymal/medullar spinal cord tumour or tumour of unspecified location within the CNS (excluding specified cranial nerve tumours)	C71<br>C72 089<br>C793*<br>D33 012479<br>D43 012479<br>Z858F	191<br>192C<br>198D*<br>225AD<br>237F	191<br>192,10 21<br>198,31*<br>225 00 30
Benign or malign meningeal tumour	C70<br>D32<br>D42	192BD<br>225CE<br>237G	192,31<br>225 20 40

* Metastasis to the central nervous system (CNS) is scored as 6 points when calculating the Charlson co-morbidity index but categorized as a lesion of the CNS

P. HIV/AIDS

	ICD 10	ICD 9	ICD 8
	B20-24<br>Z219	279K 079J V02J	-

Table S3. Data validation of register data against medical records from consecutive episodes of bacterial meningitis (n=46*).

Variable	*Level of agreement (n/N)*
Bacterial meningitis	46/46
Pathogen	46/46
Single or recurrent episode of bacterial meningitis	46/46
Age	46/46
Gender	46/46
Clinical setting	43/46†
Charlson co-morbidity (20 variables)	43/46 (917/920 data entries) ‡
Specific immunosuppression (6 variables)	45/46 (275/276 data entries) §
Severe sequelae (4 variables)	46/46 (184/184 data entries)

* 46 medical records of consecutive episodes of bacterial meningitis from Swedish secondary and tertiary care hospitals during the years 2005 to 2014 were retrived with one year of follow up data from hospital admittance † one episode with register information of admittance from a nursing home that was not noted as such in the medical records and two episodes with register information of being hospital-acquired that were registered in the medical records as community-acquired ‡ Information of hepatitis C in the medical records not registered as an ICD-code for > 5 years, information of diabetes mellitus without complications in the medical records not registered as an ICD-code for > 5 years and malignant neoplasia as an ICD-code entry that was not registered in the medical records during the hospital stay for bacterial meningitis § one episode of a patient with registry entries of both parenchymal and meningeal tumour of the central nervous system that had information of recurrent meningeal tumours in the medical records but no information of a parenchymal tumour

Table S4. Gram-negative Episodes per Pathogen and Clinical Setting, Sweden 1965–2014. *Neisseria meningitidis* and *Haemophilus influenzae* are excluded.

**Pathogen n (%)**	**Community-acquired n (%)**	**Healthcare facility-acquired n (%)**	**Post-neurosurgical, no indwelling device present n (%)**	**Post-neurosurgical, indwelling device present n (%)**
Adults (n=90)	**n=44**	**n=27**	**n=11**	**n=8**
Escherichia coli 53 (59)	28 (64)	16 (59)	5 (45)	4 (50)
Klebsiella spp. 20 (22)	8 (18)	5 (19)	3 (27)	4 (50)
Salmonella spp. 3 (3)	2 (5)	1 (4)	0 (0)	0 (0)
Pseudomonas spp. 12 (13)	5 (11)	4 (15)	3 (27)	0 (0)
Proteus spp. 2 (2)	1 (2)	1 (4)	0 (0)	0 (0)
Non-neonatal Children (n=22)	**n=13**	**n=2**	**n=2**	**n=5**
Escherichia coli 18 (82)	12 (92)	1 (50)	1 (50)	4 (80)
Klebsiella spp. 1 (5)	0 (0)	0 (0)	0 (0)	1 (20)
Salmonella spp. 1 (5)	0 (0)	1 (50)	0 (0)	0 (0)
Pseudomonas spp. 2 (9)	1 (8)	0 (0)	1 (50)	0 (0)
Proteus spp. 0 (0)	0 (0)	0 (0)	0 (0)	0 (0)
Neonates (n=34)	**n=10**	**n=24**	**n=0**	**n=0**
Escherichia coli 28 (82)	6 (60)	22 (92)	0 (0)	0 (0)
Klebsiella spp. 4 (12)	4 (40)	0 (0)	0 (0)	0 (0)
Salmonella spp. 1 (3)	0 (0)	1 (4)	0 (0)	0 (0)

Pseudomonas spp. 0 (0)	0 (0)	0 (0)	0 (0)	0 (0)
Proteus spp. 1 (3)	0 (0)	1 (4)	0 (0)	0 (0)

Table S5. Episodes per Pathogen and Clinical Setting in the Post-pneumococcal Vaccine Era, Sweden 2009–2014

**Pathogen n (%)**	**Community-acquired n (%)**	**Healthcare facility-acquired n (%)**	**Post-neurosurgical, no indwelling device present n (%)**	**Post-neurosurgical, indwelling device present n (%)**
Adults (n=1041)	**n=719**	**n=201**	**n=95**	**n=26**
H. influenzae 51 (5)	41 (6)	6 (3)	3 (3)	1 (4)
S. pneumoniae 487 (47)	388 (54)	66 (33)	31 (33)	2 (8)
N. meningitidis 109 (10)	88 (12)	15 (7)	5 (5)	1 (4)
Streptococci* 106 (10)	61 (8)	33 (16)	8 (8)	4 (15)
Gram-negatives 42 (4)	19 (3)	12 (6)	5 (5)	6 (23)
L. monocytogenes 82 (8)	49 (7)	31 (15)	1 (1)	1 (4)
Staphylococcus spp. 160 (15)	71 (10)	36 (18)	42 (44)	11 (42)
Polymicrobial 4 (0)	2 (0)	2 (1)	0 (0)	0 (0)
Non-neonatal Children (n=204)	**n=140**	**n=42**	**n=10**	**n=12**
H. influenzae 12 (6)	8 (6)	3 (7)	1 (10)	0 (0)
S. pneumoniae 63 (31)	48 (34)	15 (36)	0 (0)	0 (0)
N. meningitidis 71 (35)	60 (43)	10 (24)	1 (10)	0 (0)
Streptococci* 27 (13)	14 (10)	10 (24)	2 (20)	1 (8)
Gram-negatives 11 (5)	5 (4)	2 (5)	1 (10)	3 (25)
L. monocytogenes 2 (1)	1 (1)	1 (2)	0 (0)	0 (0)
Staphylococcus spp. 18 (9)	4 (3)	1 (2)	5 (50)	8 (67)
Polymicrobial 0 (0)	0 (0)	0 (0)	0 (0)	0 (0)
Neonates (n=77)	**n=25**	**n=51**	**n=0**	**n=1**
H. influenzae 1 (1)	0 (0)	1 (2)	0 (0)	0 (0)
S. pneumoniae 0 (0)	0 (0)	0 (0)	0 (0)	0 (0)
N. meningitidis 0 (0)	0 (0)	0 (0)	0 (0)	0 (0)
Streptococci* 52 (68)	16 (64)	36 (71)	0 (0)	0 (0)
Gram-negatives 19 (25)	7 (28)	12 (24)	0 (0)	0 (0)
L. monocytogenes 1 (1)	0 (0)	1 (2)	0 (0)	0 (0)
Staphylococcus spp. 4 (5)	2 (8)	1 (2)	0 (0)	1 (100)
Polymicrobial 0 (0)	0 (0)	0 (0)	0 (0)	0 (0)

* Non-pneumococcal streptococci

Table S6. Recurrent Episodes* per Pathogen and Clinical Setting, Sweden 1965–2014

Pathogen n (%)	Community-acquired n (%)	Healthcare facility-acquired n (%)	Post-neurosurgical, no indwelling device present n (%)	Post-neurosurgical, indwelling device present n (%)
Adults **(n=395)**	**n=322**	**n=32**	**n=19**	**n=22**
H. influenzae 58 (15)	47 (15)	4 (12)	4 (21)	3 (14)
S. pneumoniae 254 (64)	221 (69)	19 (59)	5 (26)	9 (41)
N. meningitidis 12 (3)	11 (3)	0 (0)	1 (5)	0 (0)
Streptococci† 25 (6)	16 (5)	3 (9)	2 (11)	4 (18)
Gram-negatives 3 (1)	1 (0)	1 (3)	1 (5)	0 (0)
L. monocytogenes 6 (2)	5 (2)	1 (3)	0 (0)	0 (0)
Staphylococcus spp. 28 (7)	12 (4)	4 (12)	6 (32)	6 (27)
Polymicrobial 9 (2)	9 (3)	0 (0)	0 (0)	0 (0)
Non-neonatal Children **(n=266)**	**n=223**	**n=17**	**n=8**	**n=18**
H. influenzae 48 (18)	41 (18)	4 (24)	1 (12)	2 (11)
S. pneumoniae 126 (47)	115 (52)	7 (41)	1 (12)	3 (17)
N. meningitidis 13 (5)	12 (5)	1 (6)	0 (0)	0 (0)
Streptococci† 52 (20)	43 (19)	5 (29)	3 (38)	1 (6)
Gram-negatives 5 (2)	1 (0)	0 (0)	0 (0)	4 (22)
L. monocytogenes 6 (2)	3 (1)	0 (0)	2 (25)	1 (6)
Staphylococcus spp. 14 (4)	6 (3)	0 (0)	1 (12)	7 (39)
Polymicrobial 2 (1)	2 (1)	0 (0)	0 (0)	0 (0)
Neonates **(n=9)**	**n=2**	**n=7**	**n=0**	**n=0**
H. influenzae 1 (11)	1 (50)	0 (0)	0 (0)	0 (0)
S. pneumoniae 1 (11)	0 (0)	1 (14)	0 (0)	0 (0)
N. meningitidis 0 (0)	0 (0)	0 (0)	0 (0)	0 (0)
Streptococci† 6 (67)	1 (50)	5 (71)	0 (0)	0 (0)
Gram-negatives 1 (11)	0 (0)	1 (14)	0 (0)	0 (0)
L. monocytogenes 0 (0)	0 (0)	0 (0)	0 (0)	0 (0)
Staphylococcus spp. 0 (0)	0 (0)	0 (0)	0 (0)	0 (0)
Polymicrobial 0 (0)	0 (0)	0 (0)	0 (0)	0 (0)

* In total, 670 episodes occurred in patients with recurrent bacterial meningitis and 406 were recurrent episodes following an initial episode † Non-pneumococcal streptococci

Table S7. Univariate Covariate Association of Specific Immunosuppressive Conditions with Recurrent Episodes, Sweden 1997–2014*

Several deficiencies were not specified in ICD9 (that was used before the year 1997), for example common variable immunodeficiency (CVID). To further minimize potential bias due to different versions of the International Classification of Diseases (ICD), the analysis was restricted to the period after introduction of ICD10 in the year 1997.

**Specific immunosuppressive condition (n/N)**	**Crude RR† (95 % CI)**	**P value**
Lesions of the central nervous system (65/575)	2,7 (1,9–4,0)	**<0,001**
Post-neurosurgical episode (41/338)	2,5 (1,6–3,8)	**<0,001**
No indwelling device present (20/240)	1,6 (0,9–2,8)	0,09
Indwelling device present (21/98)	3,9 (2,1–7,0)	**<0,001**
Intracranial shunt present (16/90)	3,1 (1,5–6,4)	**0,002**
Cochlear implant present (6/8)	12,8 (6,8–24,1)	**<0,001**
Intracranial electrode present (0/1)	‡	‡
Traumatic lesion (14/94)	2,5 (1,2–5,3)	**0,01**
Traumatic para-meningeal bleed (6/53)	2,2 (0,7–6,3)	0,16
Basilar skull fracture (12/54)	3,7 (1,7–8,0)	**0,001**
Open cranial fracture (2/14)	1,6 (0,3–9,6)	0,60
Neoplastic lesion, not specified as metastasis (11/108)	2,1 (1,0–4,5)	**0,05**
Parenchymal benign and malignant tumour (7/95)	1,5 (0,6–4,1)	0,41
Meningeal benign and malignant tumour (5/30)	3,8 (1,5–9,7)	**0,005**
Cerebrovascular lesion (18/244)	1,6 (0,9–2,9)	0,14
Ischemic (14/124)	3,5 (1,8–6,7)	**<0,001**
Haemorrhagic (8/149)	0,9 (0,3–2,5)	0,9
Malignant neoplasia (28/432)	2,5 (1,4–4,2)	**0,001**
Haematological malignancy (12/157)	3,2 (1,4–7,1)	**0,004**
Lymphoma (6/43)	4,6 (1,6–13,4)	**0,004**
Leukaemia (5/68)	2,8 (0,9–8,9)	0,07
Malignant plasma cell neoplasia (2/58)	2,0 (0,3–12,8)	0,48
Other malignant neoplasia (27/382)	2,5 (1,5–4,4)	**0,001**
Pharmacological immunosuppression§		
Steroid pre-treatment (8/146)	1,7 (0,8–3,8)	0,17
Other Charlson Co-morbidity (18/311)	1,7 (0,9–3,5)	0,1
Rheumatic disease (8/155)	1,3 (0,5–3,6)	0,64
Diabetes mellitus with end organ damage (3/104)	0,8 (0,2–3.4)	0,7
Moderate to severe liver disease (5/41)	4,6 (1,4–14,7)	**0,01**
Renal failure (2/41)	2,8 (0,5–17,2)	0,27
Primary immune deficiency (14/35)	12,5 (7,2–21,7)	**<0,001**

Common variable immunodeficiency (0/1)	‡	‡
Severe combined immunodeficiency (0/0)	‡	‡
Humoral deficiencies (3/7)	10,3 (5,1–20,6)	**<0,001**
Complement deficiencies (4/6)	13,7 (4,9–38,0)	**<0,001**
Monoclonal gammopathy of unknown significance (5/19)	9,4 (3,3–26,7)	**<0,001**
Other primary immune deficiencies (2/4)	16,6 (2,5–110,7)	**0,004**

* Neonates were excluded from the analysis † Relative risk for recurrent episodes as estimated with logistic regression clustered on patients and adjusted for calendar time and follow-up time ‡ Too few episodes with the current outcome § Pharmacological data available from July 2005, thus estimates are based on the number of episodes from the year 2005 to the year 2014

Table S8. Severe Sequelae per Pathogen and Clinical Setting in Single-episode Bacterial Meningitis, Sweden 2005–2014

Pharmacological data was available from the year 2005; therefore, data on severe sequelae was restricted to episodes reported from the year 2005 to the year 2014.

**Pathogen n/N (%)**	**Adults n/N (%)**	**Non-neonatal Children n/N (%)**	**Neonates n/N (%)**	**Non-neuro-surgical n/N (%)**	**Post-neuro-surgical n/N (%)**
All cause 30-day mortality					
H. influenzae 5/111 (5)	5/8 (6)	0/24 (0)	0/1 (0)	4/108 (4)	1/3 (33)
S. pneumoniae 101/905 (11)	93/744 (12)	8/160 (5)	0/1 (0)	96/869 (11)	5/36 (14)
N. meningitidis 14/286 (5)	12/167 (7)	2/119 (2)	0/0 (0)	13/279 (5)	1/7 (14)
Streptococci* 21/281 (7)	17/174 (10)	1/40 (2)	3/67 (4)	21/263 (8)	0/18 (0)
Gram-negatives 7/82 (9)	5/50 (10)	0/9 (0)	2/23 (9)	6/68 (9)	1/14 (7)
L. monocytogenes 26/122 (21)	26/119 (22)	0/2 (0)	0/1 (0)	26/120 (22)	0/2 (0)
Staphylococci† 30/287 (10)	30/254 (12)	0/29 (0)	0/4 (0)	28/184 (15)	2/103 (2)
Polymicrobial 1/10 (10)	1/8 (12)	0/2 (0)	0/0 (0)	1/10 (10)	0/0 (0)
BM‡ 205/2084 (10)	189/1602 (12)	11/385 (3)	5/97 (5)	195/1901 (10)	10/183 (5)
Severe neurological sequelae					
H. influenzae 30/111 (27)	27/86 (31)	3/24 (12)	0/1 (0)	28/108 (26)	2/3 (67)
S. pneumoniae 343/905 (38)	309/744 (42)	34/160 (21)	0/1 (0)	320/869 (37)	23/36 (64)
N. meningitidis 62/286 (22)	46/167 (28)	16/119 (13)	0/0 (0)	57/279 (20)	5/7 (71)
Streptococci* 92/281 (33)	62/174 (36)	14/40 (35)	16/67 (24)	83/263 (32)	9/18 (50)
Gram-negatives 29/82 (35)	21/50 (42)	4/9 (44)	4/23 (17)	23/68 (34)	6/14 (43)
L. monocytogenes 33/122 (27)	33/119 (28)	0/2 (0)	0/1 (0)	31/120 (26)	2/2 (100)
Staphylococci† 153/287 (53)	130/254 (51)	21/29 (72)	2/4 (50)	84/184 (46)	69/103 (67)
Polymicrobial 4/10 (40)	4/8 (50)	0/2 (0)	0/0 (0)	4/10 (40)	0/0 (0)
BM‡ 746/2084 (36)	632/1602 (39)	92/385 (24)	22/97 (23)	630/1901 (33)	116/183 (63)
Severe sequelae					
H. influenzae 34/111 (31)	31/86 (36)	3/24 (12)	0/1 (0)	32/108 (30)	2/3 (67)
S. pneumoniae 425/905 (47)	383/744 (51)	42/160 (26)	0/1 (0)	398/869 (46)	27/36 (75)
N. meningitidis 75/286 (26)	57/167 (34)	18/119 (15)	0/0 (0)	69/279 (25)	6/7 (86)
Streptococci* 109/281 (39)	76/174 (44)	15/40 (38)	18/67 (27)	100/263 (38)	9/18 (50)
Gram-negatives 36/82 (44)	26/50 (52)	4/9 (44)	6/23 (26)	29/68 (43)	7/14 (50)
L. monocytogenes 54/122 (44)	54/119 (45)	0/2 (0)	0/1 (0)	52/120 (43)	2/2 (100)
Staphylococci† 179/287 (62)	156/254 (61)	21/29 (72)	2/4 (50)	109/184 (59)	70/103 (68)
Polymicrobial 5/10 (50)	5/8 (62)	0/2 (0)	0/0 (0)	5/10 (50)	0/0 (0)

BM‡ 917/2084 (44)	788/1602 (49)	103/385 (27)	26/97 (27)	794/1901 (42)	123/183 (67)

* Non-pneumococcal streptococci † *Staphylococcus* spp. ‡ Bacterial meningitis

Table S9. Disease Burden per Pathogen and Clinical Setting in Single-episode Bacterial Meningitis, Sweden 2005–2014

Pharmacological data was available from the year 2005; therefore, data on severe sequelae was restricted to episodes reported from the year 2005 to the year 2014.

**Pathogen per episode (sum)**	**Adults per episode (sum)**	**Children per episode (sum)**	**Non-neurosurgical per episode (sum)**	**Post-neurosurgical per episode (sum)**
Years of life lost, YLL				
H. influenzae 3 (342)	4 (342)	0 (0)	3 (299)	14 (43)
S. pneumoniae 7 (6443)	9 (6422)	0 (21)	7 (6227)	6 (216)
N. meningitidis 2 (643)	4 (614)	0 (30)	2 (625)	3 (19)
Streptococci* 4 (1192)	7 (1191)	0 (1)	5 (1192)	0 (0)
Gram-negatives 5 (377)	8 (377)	0 (0)	4 (301)	5 (76)
L. monocytogenes 15 (1866)	16 (1866)	0 (0)	16 (1866)	0 (0)
Staphylococcus spp. 8 (2176)	9 (2176)	0 (0)	11 (2020)	2 (157)
Polymicrobial 8 (79)	10 (79)	0 (0)	8 (79)	0 (0)
Bacterial meningitis 6 (13 118)	8 (13 066)	0 (51)	7 (12 607)	3 (511)
Years lived with disability, YLD				
H. influenzae 3 (377)	4 (333)	2 (43)	3 (369)	2 (7)
S. pneumoniae 5 (4555)	6 (4183)	2 (372)	5 (4278)	8 (277)
N. meningitidis 2 (659)	3 (495)	1 (164)	2 (618)	6 (41)
Streptococci* 4 (1170)	6 (994)	2 (176)	4 (959)	12 (211)
Gram-negatives 4 (297)	6 (279)	1 (18)	4 (251)	3 (46)
L. monocytogenes 4 (456)	4 (456)	0 (0)	4 (449)	4 (7)
Staphylococcus spp. 6 (1761)	6 (1578)	6 (183)	5 (975)	8 (786)
Polymicrobial 11 (109)	14 (109)	0 (0)	11 (109)	0 (0)
Bacterial meningitis 5 (9384)	5 (8428)	2 (956)	4 (8009)	8 (1375)
Disability adjusted life years, DALY				
H. influenzae 6 (719)	8 (675)	2 (43)	6 (668)	17 (51)
S. pneumoniae 12 (10998)	14 (10 605)	2 (393)	12 (10 505)	14 (493)
N. meningitidis 5 (1302)	7 (1108)	2 (194)	4 (1242)	9 (60)
Streptococci* 8 (2362)	13 (2185)	2 (177)	8 (2151)	12 (211)
Gram-negatives 8 (674)	13 (656)	1 (18)	8 (552)	9 (122)

L. monocytogenes 19 (2322)	20 (2322)	0 (0)	19 (2315)	4 (7)
Staphylococcus spp. 14 (3937)	15 (3754)	6 (183)	16 (2995)	9 (942)
Polymicrobial 19 (188)	23 (188)	0 (0)	19 (188)	0 (0)
Bacterial meningitis 11 (22 502)	13 (21 494)	2 (1007)	11 (20 616)	10 (1886)

* Non-pneumococcal streptococci

Table S10. The Importance of Specific Immunosuppression for Severe Sequelae in Single-episode, Non-neurosurgical Bacterial Meningitis, Sweden years 2005 to 2014

Pharmacological data was available from the year 2005; therefore, data on severe sequelae was restricted to episodes reported episodes reported from the year 2005 to the year 2014.

Age group	OR (CI95%)	P-value	Adjusted OR* (CI95%)	P-value
**30-day all-cause mortality**				
Age 0 to 4 years (non-neonatal)	0.2 (0.1 to 0.4)	**<0.001**	1 (reference)	-
Age 5 to 17 years	0.2 (0.1 to 0.8)	**0.02**	1.1 (0.3 to 4.5)	0.89
Age 18 to 64 years	0.6 (0.4 to 0.8)	**<0.001**	**3.1 (1.3 to 7.3)**	**0.009**
Age over 65 years	3.9 (2.8 to 5.3)	**<0.001**	**8.7 (3.7 to 20.3)**	**<0.001**
Male gender	1.0 (0.8 to 1.4)	0.91	1.0 (0.7 to 1.3)	0.89
Healthcare facility-acquired	1.3 (0.9 to 1.8)	0.17	1.3 (0.9 to 1.9)	0.13
Specific immunosuppression	**1.8 (1.3 to 2.4)**	**<0.001**	-	-
Lesions of the CNS†	0.9 (0.4 to 1.6)	0.62	0.6 (0.3 to 1.2)	0.14
Malignant neoplasia‡	2.5 (1.7 to 3.6)	**<0.001**	**1.6 (1.1 to 2.4)**	**0.02**
Steroid treatment§	2.1 (1.3 to 3.4)	**0.003**	1.2 (0.7 to 2.0)	0.55
Other Charlson co-morbidities‖	1.4 (0.9 to 2.2)	0.12	0.9 (0.6 to 1.5)	0.77
Primary immune deficiency	0.8 (0.1 to 6.2)	0.83	0.4 (0.1 to 3.)	0.41
Neonate**	0.5 (0.2 to 1.2)	0.10	1.9 (0.6 to 6.6)	0.29
**Severe neurological sequelae**				
Age 0 to 4 years (non-neonatal)	**0.5 (0.3 to 0.6)**	**<0.001**	1 (reference)	-
Age 5 to 17 year	**0.5 (0.3 to 0.8)**	**0.004**	1.1 (0.6 to 1.8)	0.85
Age 18 to 64 years	**1.4 (1.1 to 1.7)**	**0.001**	2.7 (1.9 to 3.7)	**<0.001**
Age over 65 years	1.3 (1.0 to 1.6)	**0.02**	4.1 (2.9 to 5.8)	**<0.001**
Male gender	1.0 (0.8 to 1.2)	0.90	0.9 (0.8 to 1.3)	0.47
Healthcare facility-acquired	0.9 (0.7 to 1.1)	0.28	1.0 (0.8 to 1.3)	0.76
Specific immunosuppression	1.0 (0.8 to 1.2)	0.88	-	-
Lesions of the CNS†	**1.7 (1.2 to 2.4)**	**0.005**	1.4 (1.0 to 2.1)	0.06
Malignant neoplasia‡	0.8 (0.6 to 1.1)	0.13	0.8 (0.6 to 1.1)	0.15
Steroid treatment§	0.8 (0.5 to 1.2)	0.34	0.9 (0.6 to 1.3)	0.60
Other Charlson co-morbidities‖	1.0 (0.8 to 1.4)	0.83	0.9 (0.6 to 1.2)	0.48

Primary immune deficiency	1.0 (0.3 to 3.3)	0.99	0.8 (0.2 to 2.6)	0.71
Neonate**	**0.6 (0.4 to 1.0)**	**0.031**	1.3 (0.7 to 2.2)	0.41
**Severe sequelae**				
Age 0 to 4 years (non-neonatal)	0.4 (0.3 to 0.5)	**<0.001**	1 (reference)	-
Age 5 to 17 years	0.4 (0.3 to 0.6)	**<0.001**	1.1 (0.6 to 1.8)	0.85
Age 18 to 64 years	1.1 (0.9 to 1.4)	0.21	2.7 (1.9 to 3.7)	**<0.001**
Age over 65 years	1.9 (1.6 to 2.4)	**<0.001**	4.1 (2.9 to 5.8)	**<0.001**
Male gender	1.0 (0.8 to 1.2)	0.80	0.9 (0.8 to 1.1)	0.47
Healthcare facility-acquired	1.0 (0.8 to 1.2)	0.88	1.0 (0.8 to 1.3)	0.76
Specific immunosuppression	**1.2 (1.0 to 1.5)**	**0.04**	-	-
Lesions of the CNS†	**1.7 (1.2 to 2.4)**	**0.006**	1.4 (1.0 to 2.1)	0.06
Malignant neoplasia‡	1.1 (0.9 to 1.5)	0.34	0.8 (0.6 to 1.1)	0.15
Steroid treatment§	1.1 (0.8 to 1.6)	0.58	0.9 (0.6 to 1.3)	0.60
Other Charlson co-morbidities‖	1.2 (0.9 to 1.6)	0.29	0.9 (0.6 to 1.2)	0.48
Primary immune deficiency	1.0 (0.3 to 3.1)	0.99	0.8 (0.2 to 2.6)	0.71
Neonate**	**0.5 (0.3 to 0.8)**	**0.003**	1.3 (0.7 to 2.2)	0.41

* Odds ratio estimated with multivariate logistic regression adjusted for healthcare facility-acquired bacterial meningitis (using community-acquired bacterial meningitis as reference), specific immunosuppression and age groups † Including traumatic, neoplastic and cerebrovascular lesions of the central nervous system ‡ Including haematological and non-haematological forms § Systemic treatment ongoing for at least two weeks and reported within one year of admission ‖ Rheumatic disease, diabetes mellitus with end organ damage, moderate to severe liver disease and renal failure ** Age < 28 days

SUPPLEMENTARY FIGURES

Figure S1. Incidence Rate per Clinical Setting of Bacterial Meningitis in Sweden 1995 to 2014.

The incidence rate of community-acquired (CBM), non-neurosurgical healthcare facility-acquired (HBMF) and post-neurosurgical (PBM) episodes of bacterial meningitis are shown per pathogen on the y–axis. **(A)** All bacteria, **(B)** *H. influenzae*, **(C)** *S. pneumoniae*, **(D)** *N. meningitidis* **(E)** Non-pneumococcal streptococci, **(F)** Gram-negative species, **(G)** *L. monocytogenes*, **(H)** Staphylococcus species. Predicted estimates with a relative standard error of >0.3 and confidence intervals with negative values were suppressed. Predicted incidence rates per clinical setting are depicted as median splines. Py denotes person years.

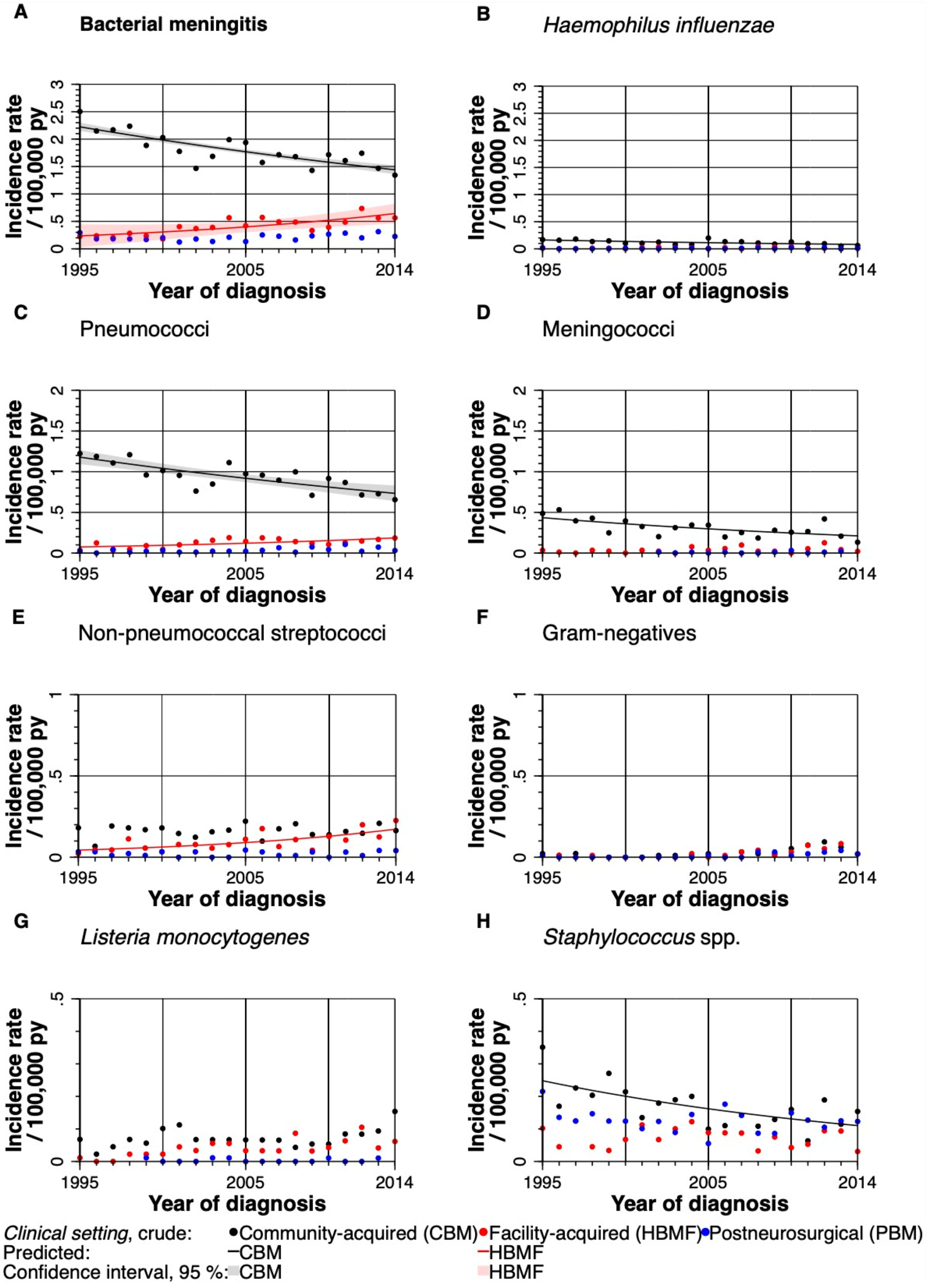
A
Bacterial meningitis
B
Haemophilus influenzae
C
Pneumococci
D
Meningococci
E
Non-pneumococcal streptococci
F
Gram-negatives
G
Listeria monocytogenes
H
Staphylococcus spp.
Incidence rate / 100,000 py
Year of diagnosis
1995
2005
2014
0
.5
1
1.5
2
2.5
3
Clinical setting, crude: Community-acquired (CBM) Facility-acquired (HBMF) Postneurosurgical (PBM)
Predicted: CBM HBMF
Confidence interval, 95 %: CBM HBMF

Figure S2. Crude Incidence Rate and Proportions of Episodes per Clinical Setting in the Conjugate Vaccine Era, Sweden 1995 to 2014.

The scatter plot shows the annual incidence rate (IR) of bacterial meningitis (BM) in Sweden during the conjugate vaccine era (years 1995 to 2014) per clinical setting on the left y–axis (y1) and annual stacked proportions of clinical settings including the proportion that was of neonatal age and/or had an indwelling intracranial device present at admission on the right y–axis (y2). The annual total number of episodes are shown above for each year. **(A)** all bacterial species, **(B)** *Haemophilus influenzae*, **(C)** *Streptococcus pneumoniae*, **(D)** *Neisseria meningitidis*, **(E)** streptococci (non-pneumococcal streptococci), **(F)** Gram-negatives (except *Neisseria meningitidis* and *Haemophilus influenzae*), **(G)** *Listeria monocytogenes*, **(H)** *Staphylococcu*s species. Py denotes person years. PCV denotes pneumococcal conjugate vaccine. CBM denotes community-acquired BM. HBMF denotes non-neurosurgical healthcare facility-acquired BM, a form of healthcare-associated BM. PBM denotes post-neurosurgical BM, a form of healthcare-associated BM that may occur with or without an indwelling intracranial device present at admission.

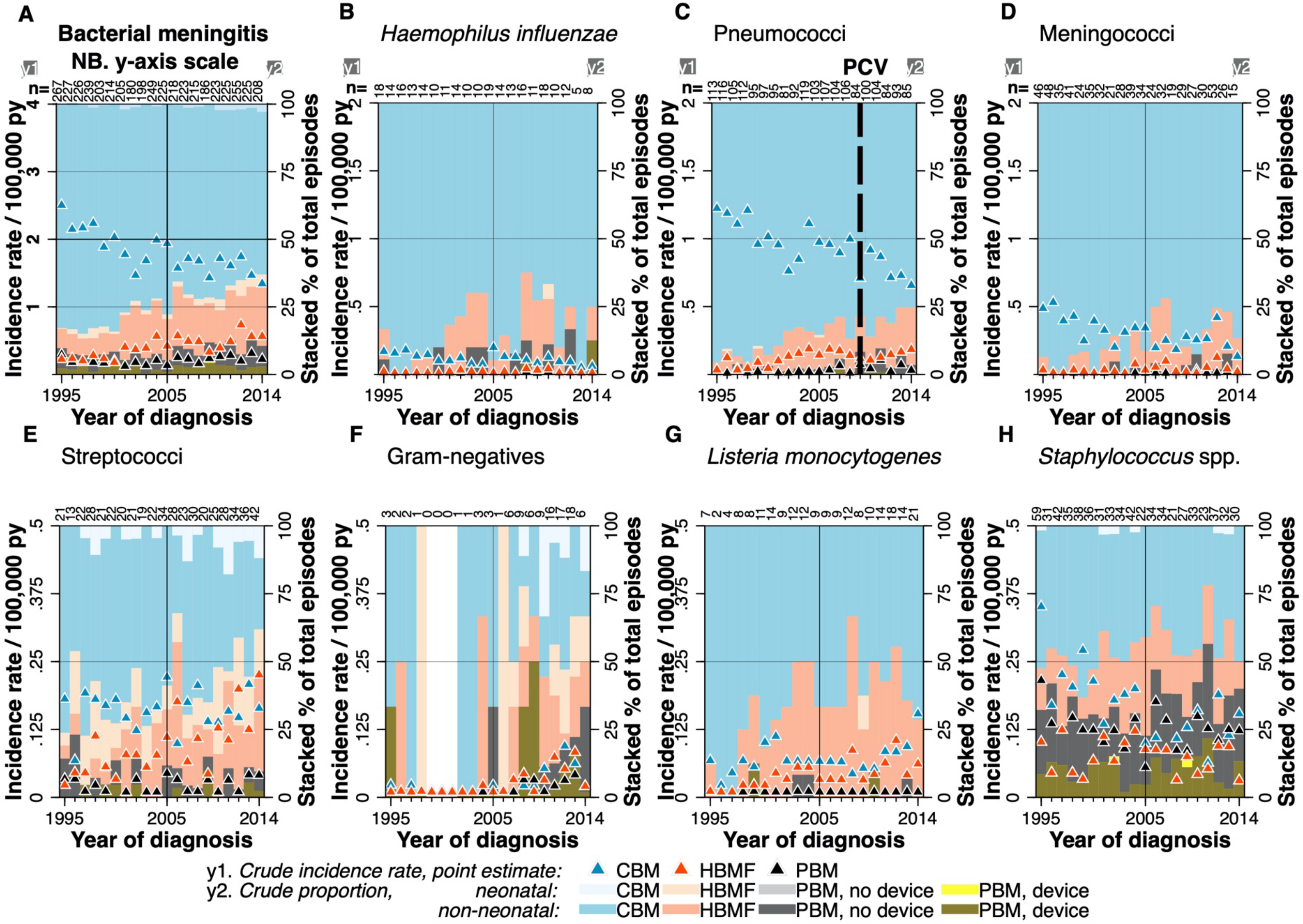
A
Bacterial meningitis
NB. y-axis scale
B
Haemophilus influenzae
C
Pneumococci
PCV
D
Meningococci
E
Streptococci
F
Gram-negatives
G
Listeria monocytogenes
H
Staphylococcus spp.
y1
y2
n=
Incidence rate / 100,000 py
Stacked % of total episodes
Year of diagnosis
1995
2005
2014
y1. Crude incidence rate, point estimate: CBM HBMF PBM
y2. Crude proportion, neonatal: CBM HBMF PBM, no device PBM, device
non-neonatal: CBM HBMF PBM, no device PBM, device

Figure S3. Median Patient Age and the Incidence Rate of Neonatal Bacterial Meningitis in the Conjugate-vaccine Era, Sweden 1995 to 2014

(**A-C**) shows the crude and predicted annual median age at admission among (A) adults, (B) non-neonatal children and (C) neonates with BM during the conjugate vaccine era together with the median age of patients post-PCV (years 2009 to 2014). (**D**) shows the crude and predicted IR (estimates with a relative standard error of >0.3 were suppressed) of neonatal BM overall and per pathogen. Py denotes person years. CI95% denotes 95 percent confidence interval. IQR denotes interquartile range. Predictions are depicted as median splines.

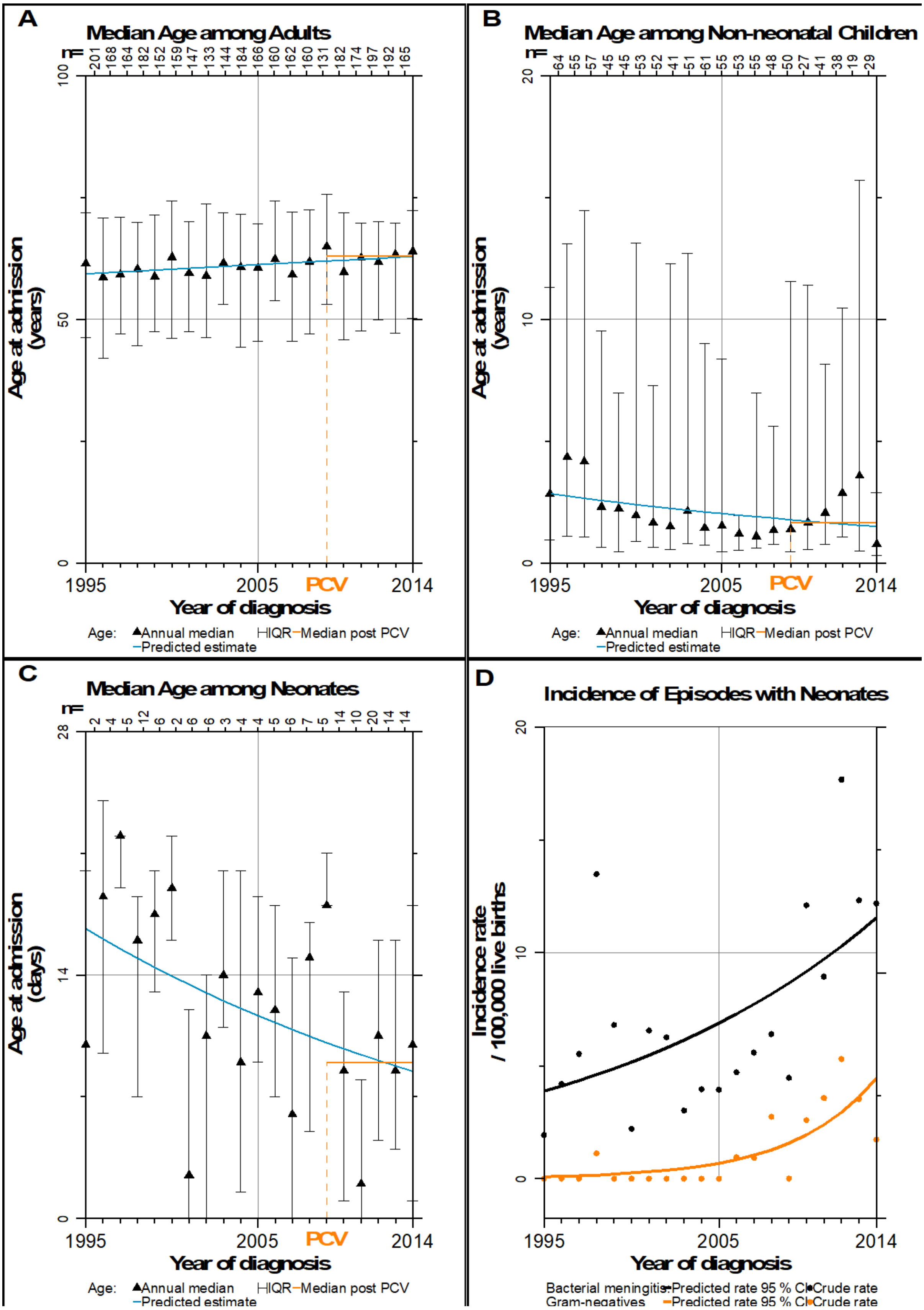
A
Median Age among Adults
n=
201 168 164 182 152 159 147 133 144 184 166 160 162 160 131 182 174 197 192 165
Age at admission (years)
100
50
0
1995
2005
PCV
2014
Year of diagnosis
Age: Annual median IQR Median post PCV
Predicted estimate
B
Median Age among Non-neonatal Children
n=
64 55 57 45 45 53 52 41 51 61 55 53 55 48 50 27 41 38 19 29
Age at admission (years)
20
10
0
1995
2005
PCV
2014
Year of diagnosis
Age: Annual median IQR Median post PCV
Predicted estimate
C
Median Age among Neonates
n=
2 4 5 12 6 2 6 6 3 4 4 5 6 7 5 14 10 20 14 14
Age at admission (days)
28
14
0
1995
2005
PCV
2014
Year of diagnosis
Age: Annual median IQR Median post PCV
Predicted estimate
D
Incidence of Episodes with Neonates
Incidence rate / 100,000 live births
20
10
0
1995
2005
2014
Year of diagnosis
Bacterial meningitis Predicted rate 95 % CI Crude rate
Gram-negatives Predicted rate 95 % CI Crude rate

Figure S4. Specific Immunosuppression of Patients with Bacterial Meningitis

(A) Pathogen specific profiles of specific immunosuppressive conditions and high age among adult community-acquired episodes compared to adult non-neurosurgical facility-acquired episodes post-PCV (years 2009 to 2014). **(B)** Pathogen specific profiles of rheumatic disease among adult community-acquired compared to adult non-neurosurgical facility-acquired episodes post-PCV. **(C and D)** Pathogen specific profiles of malignancies and non-neoplastic lesions of the central nervous system (CNS) post-PCV. Traumatic lesions of the central nervous system include basilar skull fracture, compound skull fracture and traumatic para-meningeal bleed. **(E-H)** shows the corresponding estimates as in A-D for children. Post-neurosurgical episodes are excluded as they are per definition preceded by a lesion of the blood-brain barrier, a specific form of immunosuppression. *H. influenzae* denotes *Haemophilus influenzae*. *L. monocytogenes* denotes *Listeria monocytogenes*. Streptococci denotes non-pneumococcal streptococci. Significance levels are shown as $p<0.05$ (*), $p<0.01$ (**), $p<0.001$ (***) and non-significant (NS).

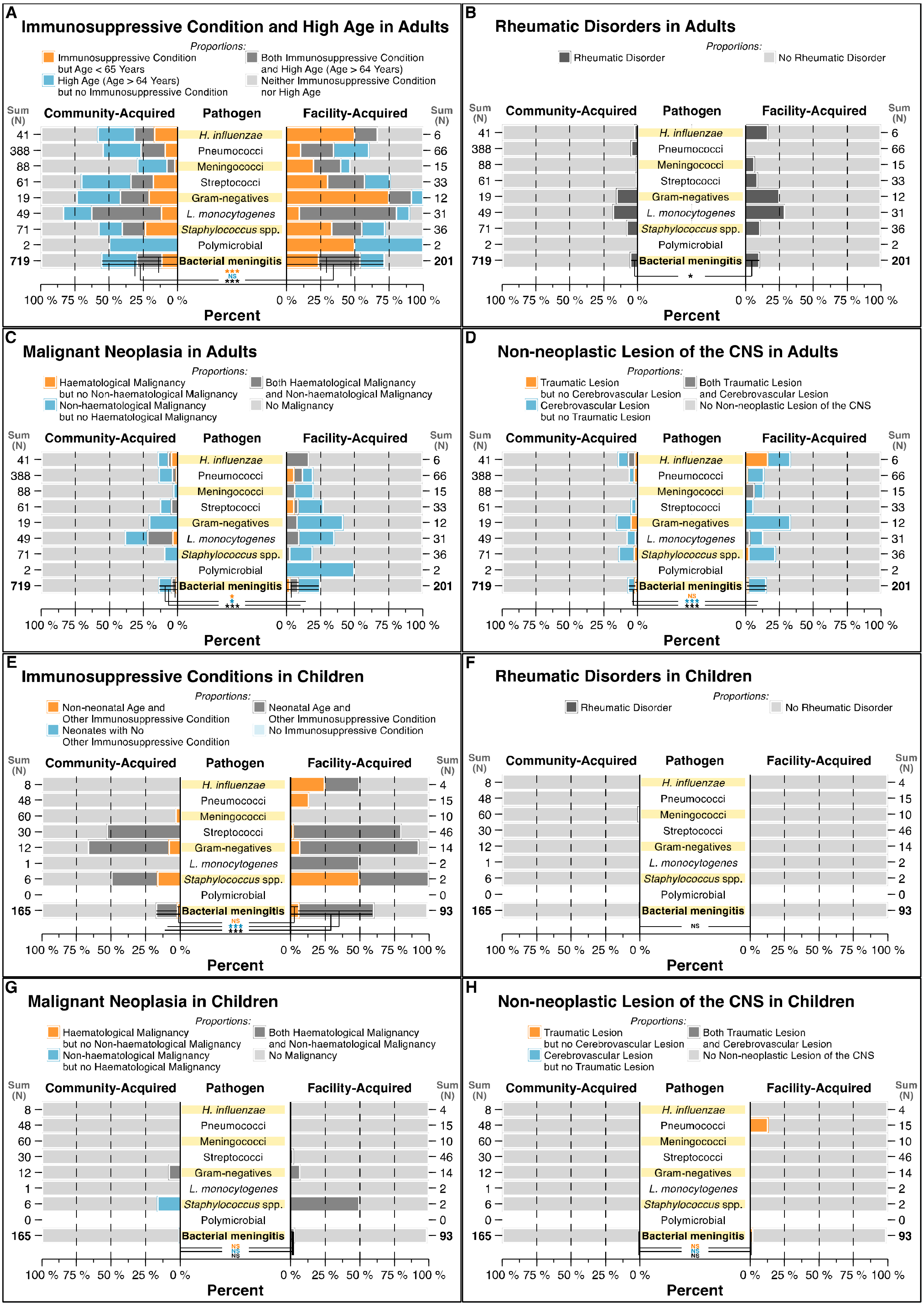
A
Immunosuppressive Condition and High Age in Adults
Proportions:
Immunosuppressive Condition but Age < 65 Years
High Age (Age > 64 Years) but no Immunosuppressive Condition
Both Immunosuppressive Condition and High Age (Age > 64 Years)
Neither Immunosuppressive Condition nor High Age
B
Rheumatic Disorders in Adults
Proportions:
Rheumatic Disorder
No Rheumatic Disorder
C
Malignant Neoplasia in Adults
Proportions:
Haematological Malignancy but no Non-haematological Malignancy
Non-haematological Malignancy but no Haematological Malignancy
Both Haematological Malignancy and Non-haematological Malignancy
No Malignancy
D
Non-neoplastic Lesion of the CNS in Adults
Proportions:
Traumatic Lesion but no Cerebrovascular Lesion
Cerebrovascular Lesion but no Traumatic Lesion
Both Traumatic Lesion and Cerebrovascular Lesion
No Non-neoplastic Lesion of the CNS
E
Immunosuppressive Conditions in Children
Proportions:
Non-neonatal Age and Other Immunosuppressive Condition
Neonates with No Other Immunosuppressive Condition
Neonatal Age and Other Immunosuppressive Condition
No Immunosuppressive Condition
F
Rheumatic Disorders in Children
Proportions:
Rheumatic Disorder
No Rheumatic Disorder
G
Malignant Neoplasia in Children
Proportions:
Haematological Malignancy but no Non-haematological Malignancy
Non-haematological Malignancy but no Haematological Malignancy
Both Haematological Malignancy and Non-haematological Malignancy
No Malignancy
H
Non-neoplastic Lesion of the CNS in Children
Proportions:
Traumatic Lesion but no Cerebrovascular Lesion
Cerebrovascular Lesion but no Traumatic Lesion
Both Traumatic Lesion and Cerebrovascular Lesion
No Non-neoplastic Lesion of the CNS
Sum (N)
Community-Acquired
Pathogen
Facility-Acquired
H. influenzae
Pneumococci
Meningococci
Streptococci
Gram-negatives
L. monocytogenes
Staphylococcus spp.
Polymicrobial
Bacterial meningitis
Adults: 41, 388, 88, 61, 19, 49, 71, 2, 719 | 6, 66, 15, 33, 12, 31, 36, 2, 201
Children: 8, 48, 60, 30, 12, 1, 6, 0, 165 | 4, 15, 10, 46, 14, 2, 2, 0, 93
100 % 75 % 50 % 25 % 0 %
0 % 25 % 50 % 75 % 100 %
Percent
NS

Figure S5. Distribution of Single and Multiple Severe Neurological Sequelae, Sweden 2005 to 2014.

The overall frequency (N=746/2084 single episodes of bacterial meningitis) of different neurological sequelae forms is shown adjacent to each coloured field; (**blue**) non-structural forms, (**red**) cerebrovascular lesions, (**green**) hydrocephalus, (**yellow**) non-psychological neurological and (**grey**) psychological. The distribution of single (coloured fields without overlap) and multiple (overlapping coloured fields) severe neurological sequelae forms are shown within the respective fields (n). Categories within the coloured circles are mutually exclusive.

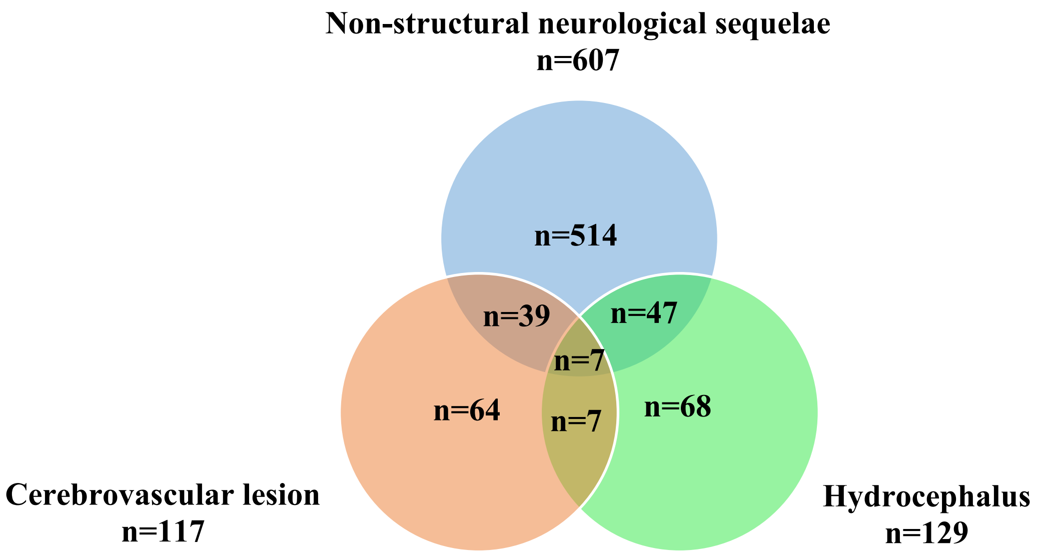

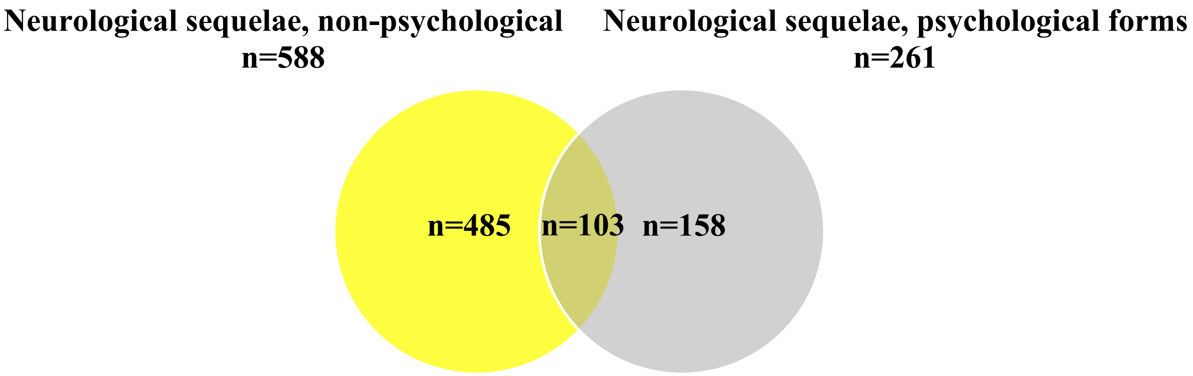

Figure S6. Mortality Within 30 Days of Hospital Admission with Bacterial Meningitis

The scatter plot shows the annual case fatality rate (CFR) in Sweden during the years 1965 to 2014 per pathogen and age group in relation to introduction of the *H. influenzae B* (Hib) and pneumococcal conjugate vaccine (PCV). Fitted lines in colour show the predicted trend of CFR during the period with national coverage in the Inpatient Register (1987 to 2014). The 30-day mortality among patients with RBM was 5% and 2% of RBM-episodes were fatal. Haemophilus denotes *Haemophilus influenzae*, Listeria denotes *Listeria monocytogenes,* staphylococci denote *staphylococcus* spp. and streptococci denotes non-pneumococcal streptococci. Predicted estimates with a relative standard error of >0.3 were suppressed.

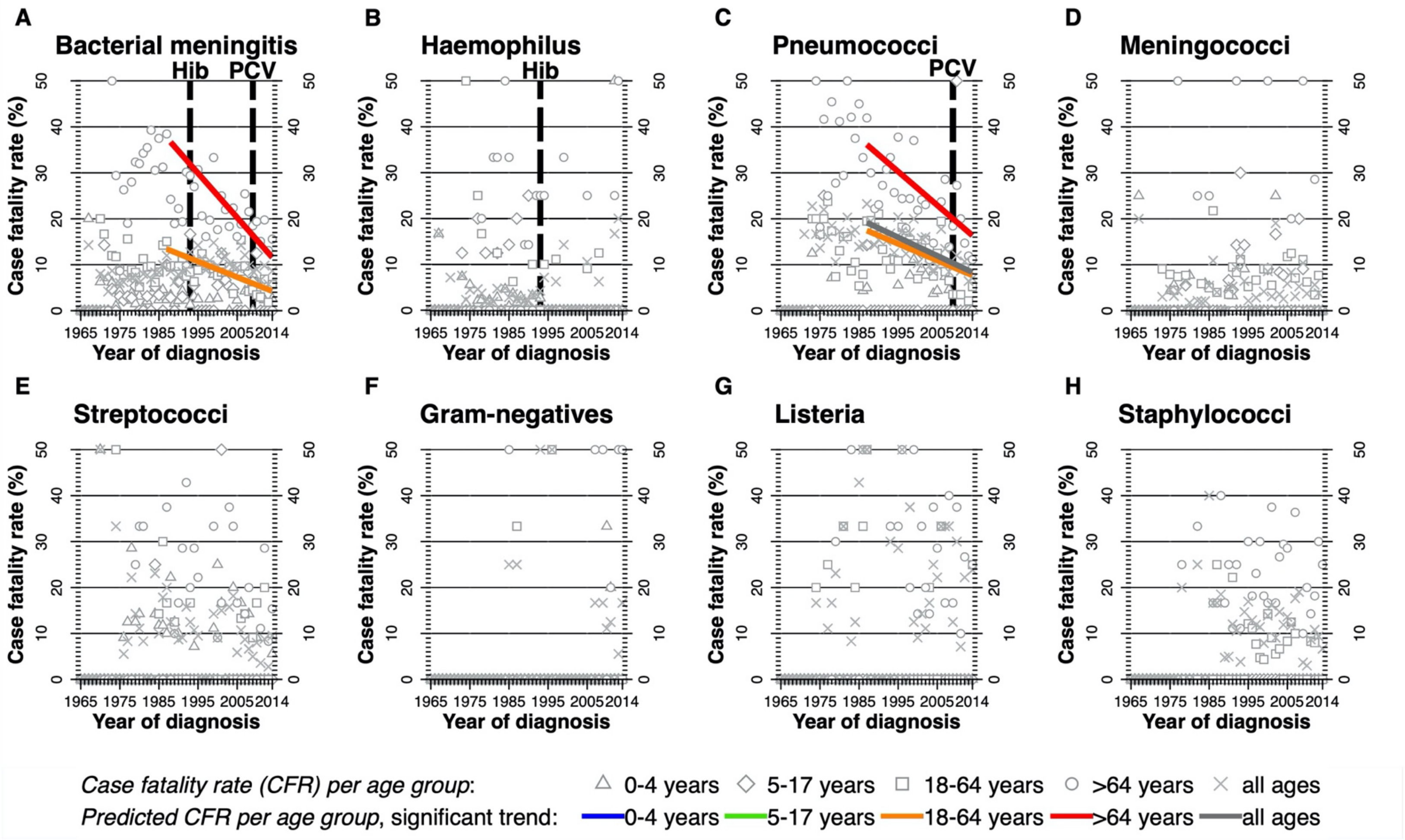
A
Bacterial meningitis
Hib
PCV
B
Haemophilus
Hib
C
Pneumococci
PCV
D
Meningococci
E
Streptococci
F
Gram-negatives
G
Listeria
H
Staphylococci
Case fatality rate (%)
Year of diagnosis
0
10
20
30
40
50
1965 1975 1985 1995 2005 2014
Case fatality rate (CFR) per age group:
0-4 years
5-17 years
18-64 years
>64 years
all ages
Predicted CFR per age group, significant trend:
0-4 years
5-17 years
18-64 years
>64 years
all ages

Figure S7. Mortality within 30 Days of Hospital Admission with Bacterial Meningitis

The scatter plot shows the annual case fatality rate (CFR) in Sweden during the years 1965 to 2014 per clinical setting in relation to introduction of the *H. influenzae B* (Hib) and pneumococcal conjugate vaccine (PCV). Fitted lines in colour show the predicted trend of CFR during the period with national coverage in the Inpatient Register (1987 to 2014). CBM denotes community-acquired bacterial meningitis. HBMF denotes non-neurosurgical healthcare facility-acquired bacterial meningitis. PBM denotes post-neurosurgical bacterial meningitis. Haemophilus denotes *Haemophilus influenzae*, Listeria denotes *Listeria monocytogenes,* staphylococci denote *staphylococcus* spp. and streptococci denotes non-pneumococcal streptococci. Predicted estimates with a relative standard error of >0.3 were suppressed.

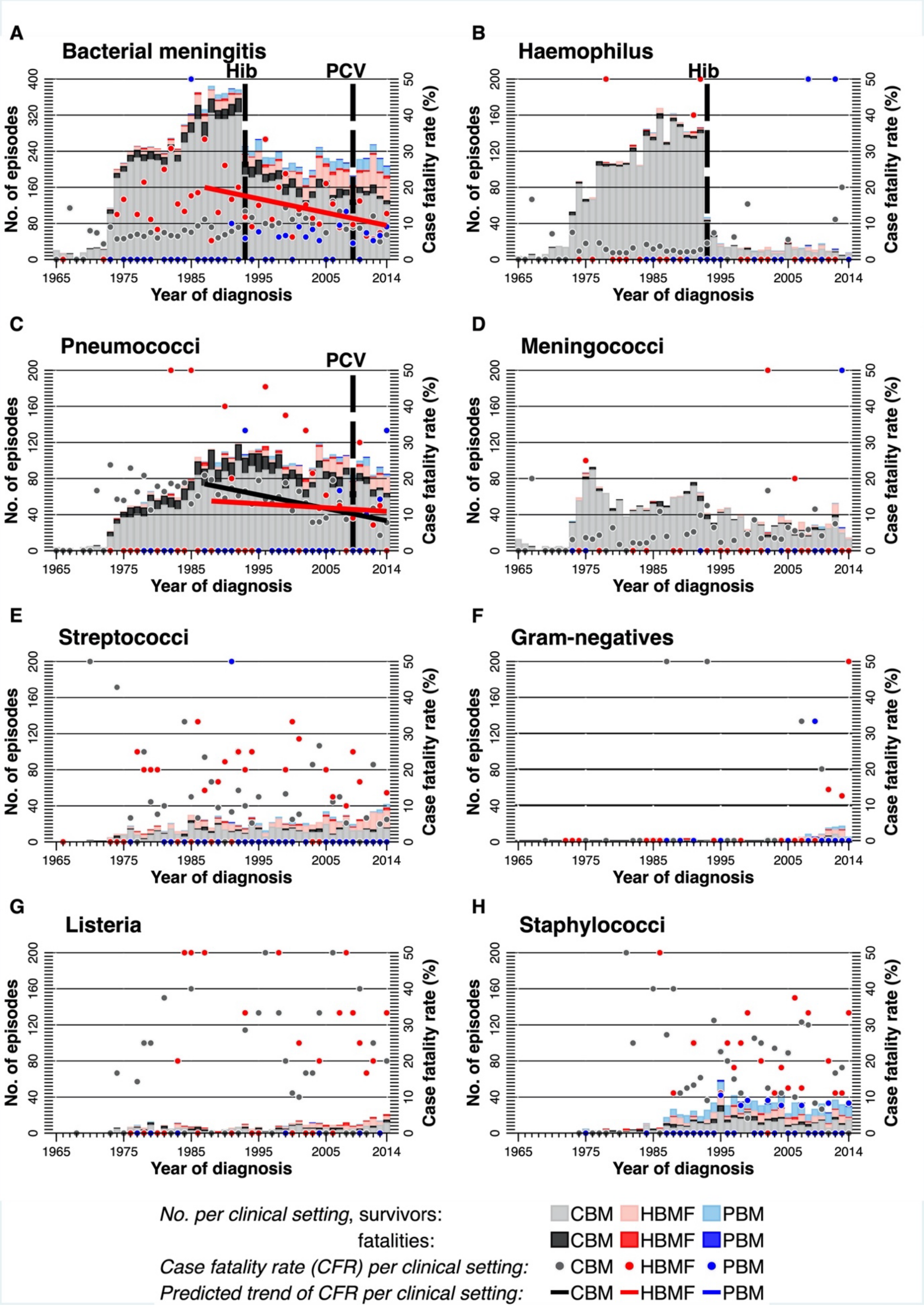

A
Bacterial meningitis
Hib
PCV
B
Haemophilus
Hib
C
Pneumococci
PCV
D
Meningococci
E
Streptococci
F
Gram-negatives
G
Listeria
H
Staphylococci
No. of episodes
Case fatality rate (%)
Year of diagnosis
No. per clinical setting, survivors: CBM HBMF PBM
fatalities: CBM HBMF PBM
Case fatality rate (CFR) per clinical setting: CBM HBMF PBM
Predicted trend of CFR per clinical setting: CBM HBMF PBM

References

1. Tebruegge M, Curtis N. Epidemiology, etiology, pathogenesis, and diagnosis of recurrent bacterial meningitis. Clin Microbiol Rev. 2008;21(3):519-37.
2. Reefhuis J, Honein MA, Whitney CG, Chamany S, Mann EA, Biernath KR, et al. Risk of bacterial meningitis in children with cochlear implants. N Engl J Med. 2003;349(5):435-45.
3. Qureshi A, Quinones-Hinojosa A, Ziai W. A rare infectious presentation of a temporal bone meningioma. J Neurooncol. 2014;116(3):633-4.
4. Picard C, Puel A, Bustamante J, Ku CL, Casanova JL. Primary immunodeficiencies associated with pneumococcal disease. Curr Opin Allergy Clin Immunol. 2003;3(6):451-9.
5. Sjoholm AG, Braconier JH, Soderstrom C. Properdin deficiency in a family with fulminant meningococcal infections. Clin Exp Immunol. 1982;50(2):291-7.
6. Kadry H, Noorani B, Cucullo L. A blood-brain barrier overview on structure, function, impairment, and biomarkers of integrity. Fluids Barriers CNS. 2020;17(1):69.
7. Keep RF, Zhou N, Xiang J, Andjelkovic AV, Hua Y, Xi G. Vascular disruption and blood-brain barrier dysfunction in intracerebral hemorrhage. Fluids Barriers CNS. 2014;11:18.
8. Jiang X, Andjelkovic AV, Zhu L, Yang T, Bennett MVL, Chen J, et al. Blood-brain barrier dysfunction and recovery after ischemic stroke. Prog Neurobiol. 2018;163-164:144-71.
